# Micro- and Nanoplastics as Emerging Drivers of Liver Injury: Exposure, Evidence, and Mechanisms

**DOI:** 10.3390/ijms27125187

**Published:** 2026-06-08

**Authors:** Miłosz Badach, Jakub Banaszek, Kinga Barańska, Jakub Kleinrok, Michał Flieger, Jolanta Flieger, Grzegorz Teresiński, Alicja Forma, Ryszard Sitarz, Jacek Baj

**Affiliations:** 1 Department of Forensic Medicine, Medical University of Lublin, Jaczewskiego 8b, 20-090 Lublin, Poland; mimbad@interia.pl (M.B.); kubabanaszek40@gmail.com (J.B.); kinga14b@gmail.com (K.B.); michalflieeeger@gmail.com (M.F.); grzegorz.teresinski@umlub.edu.pl (G.T.); 2 Department of Clinical Pathomorphology, Medical University of Lublin, Jaczewskiego 8b, 20-090 Lublin, Poland; klejs.90@gmail.com; 3 Doctoral School, Medical University of Lublin, Aleje Racławickie 1, 20-059 Lublin, Poland; 4 Department of Analytical Chemistry, Medical University of Lublin, Chodźki 4a (Collegium Pharmaceuticum), 20-093 Lublin, Poland; jolanta.flieger@umlub.edu.pl; 5 1st Department of Psychiatry, Psychotherapy and Early Intervention, Medical University of Lublin, Gluska Street 1, 20-439 Lublin, Poland; ryszard.sitarz@umlub.edu.pl; 6 Department of Correct, Clinical and Imaging Anatomy, Medical University of Lublin, Jaczewskiego 4, 20-090 Lublin, Poland

**Keywords:** microplastics, nanoplastics, MNPs, liver injury, hepatotoxicity, oxidative stress, inflammation

## Abstract

Micro- and nanoplastics (MNPs) are emerging environmental contaminants of increasing relevance to human health. Growing evidence suggests that, following ingestion, inhalation, or, less convincingly, dermal exposure, MNPs may cross biological barriers, enter lymphatic and vascular compartments, and reach the liver. Owing to portal blood flow, sinusoidal architecture and Kupffer cell activity, the liver appears to be one of the principal sites of early particle sequestration. Human biomonitoring, ex vivo and postmortem studies have detected MNPs in blood and multiple organs, including the liver, although the currently available evidence remains limited and methodologically heterogeneous. Their identification relies on multistep analytical procedures that integrate sample pretreatment with FTIR, Raman spectroscopy, LD-IR, Py-GC-MS and supplementary imaging methods. However, each of these techniques presents significant limitations, particularly in the analysis of nanoplastics. Experimental studies indicate that MNPs may induce hepatic injury through oxidative stress, mitochondrial impairment, endoplasmic reticulum stress, inflammation, DNA damage, dysregulated lipid metabolism and disruption of the gut–liver axis, consequently contributing to steatosis, cholestatic anomalies and fibrosis. Consequently, MNPs should be considered potential contributors to liver pathology, although more comprehensive human data are still required.

## 1. Introduction

### 1.1. Definitions

Microplastics (MPs) and nanoplastics (NPs), collectively referred to as micro- and nanoplastics (MNPs), are categorized as plastic particles. MPs are defined as synthetic polymer particles that are insoluble in water, exhibit either regular or irregular shapes and range in size from 1 μm to 5 mm [1]. NPs, according to current definitions, include particles with dimensions between 1 and 1000 nm [2].

### 1.2. Classification of MNPs

Based on their origin, MNPs are classified as primary or secondary. Primary MPs are intentionally manufactured as small particles and are used, for example, as additives in cosmetics (e.g., exfoliating microbeads), paints, pharmaceuticals and diapers [3,4]. In contrast, secondary MPs are formed as a result of the breakdown of larger plastic items such as fishing nets, plastic bags [4], bottles, clothing, tires and packaging materials [5]. This process occurs under the influence of physical, chemical, and biological factors, such as ultraviolet (UV) lighting [3]. The factors listed above also lead to the aging of MPs present in the environment. Aged microplastics (aMPs) are characterized by more diverse morphology and greater surface roughness, which is associated with a greater ability to adsorb chemicals and an enhanced toxic potential. Consequently, this may lead to increased oxidative stress, inflammatory responses and disruptions in the microbiota and metabolic processes [6]. NPs can be produced intentionally or formed secondarily as a result of the degradation of larger plastics or MPs [7].

MNPs can also be classified based on their chemical composition. The most commonly identified particles are composed of polyethylene (PE), polystyrene (PS), polypropylene (PP), polyurethane (PU), polyvinyl chloride (PVC) and polyethylene terephthalate (PET) [4,7]. MPs are characterized by high durability and a very slow degradation rate [4]. MNPs spread easily in the environment, as they can be transported to surface waters, carried through the air and deposited with precipitation. Furthermore, they can be ingested by aquatic organisms and undergo bioaccumulation [4,8].

### 1.3. Biological Activity of MNPs

The assessment of MNP toxicity in organisms is complex. It is influenced by many factors, such as particle size, shape and polymer type, as well as the species of the exposed organism, concentration and exposure conditions [9]. MNPs also contain thousands of plastic-associated chemical compounds. The main groups of additives include flame retardants, plasticizers and antioxidants. Examples of these compounds include phthalates, polybrominated diphenyl ethers (PBDEs), bisphenol A (BPA), nonylphenol, and octylphenol [10]. These substances may migrate from MNPs into environmental matrices and the human food chain, thereby increasing exposure risk and potential adverse health effects [8,11].

Furthermore, it has been demonstrated that, once released into the environment, MNPs can adsorb, accumulate and transport a variety of pollutants, such as heavy metals, toxic compounds and persistent organic pollutants (POPs), as well as antibiotics present in the environment. This ability stems from their large specific surface area and hydrophobic nature [10,11,12]. MPs also provide a surface conducive to the formation of biofilms, which are structures made up of micro-organisms adhering to their surface. Biofilm formation can lead to changes in the physical and chemical properties of these compounds [8,10]. These changes may potentially increase the toxicity and bioavailability of MPs and affect their behavior in the environment.

NPs are considered to pose an even greater risk because they exhibit higher reactivity and toxicity than MPs. Their small size facilitates cellular uptake, tissue penetration and distribution to distant organs, which may increase their harmful potential compared to MPs [4,9]. Additionally, their large surface area promotes the release of greater amounts of toxic substances [12]. Due to their ability to adsorb various contaminants, MNPs act as effective carriers of environmental pollutants present in air, drinking water and food, resulting in continuous exposure through multiple routes [8,11]. Another significant phenomenon is the fragmentation of plastics into increasingly smaller particles, which increases the risk of exposure for organisms and facilitates their entry into the body. Once inside the body, MNPs can interact with cells and membrane receptors. Both the MNPs themselves and the chemical additives and adsorbed environmental contaminants associated with them can act as agents causing adverse health effects. Their mechanisms of action include, among others, the induction of DNA damage and the activation of inflammatory responses and oxidative stress [11,13].

### 1.4. Routes of Exposure to MNPs

The primary routes of human exposure to MNPs are believed to be inhalation, ingestion and dermal contact [14]. The impact of MNPs present in outdoor air is generally considered lower than that of indoor air, due to greater air circulation outdoors. Although the exact number of inhaled particles remains uncertain, continuous exposure through inhalation is well established [14]. MNPs have been detected in many food products, including table salt, honey, seafood and mineral water [5], as well as in beverages such as beer, tea, soft drinks and milk [14]. Human skin may be directly exposed to MNPs through their presence in personal care products, such as facial cleansers, sunscreen, toothpaste, and hand soap [13].

### 1.5. The Distribution of the MNPs in the Body

There are marked differences in MNPs concentrations between different organs, which is due to the way MNPs enter the body. For this reason, higher levels of MNPs have been reported in organs directly associated with the digestive and respiratory systems. Additionally, differences in their concentrations are attributed to variations in the permeability of biological barriers or the health status of specific organs in the body [15]. MNPs inhaled with the air settle in the respiratory tract [16]. Most larger MPs particles are captured by the mucociliary clearance mechanism. Smaller particles, ranging from 1 to 5 µm in size, settle in the smaller bronchi, while NPs may penetrate deeper into the lungs and potentially cross the respiratory barrier [15]. When ingested with contaminated food and water, MPs enter the intestines, where larger MPs are more likely to remain in the intestinal lumen and are excreted in the feces, while particularly small particles, such as NPs, can penetrate the intestinal epithelium [16].

MNPs that enter the body through the skin utilize both intercellular and transcellular pathways and can penetrate through sweat glands, hair follicles, or skin lesions. Once they have crossed the skin barrier, the particles can reach the dermis and then enter the bloodstream [15]. After crossing respiratory, intestinal or skin barriers, MNPs can subsequently reach the bloodstream, from where they are transported to other organs, including the liver, spleen, brain and kidneys [15,16].

### 1.6. MNPs in the Human Body

Markers of MNPs have been detected in human urine, feces [5], sputum, bronchoalveolar lavage fluid, saliva and blood [4]. Additionally, their presence has been documented in various body fluids (e.g., pericardial fluid, synovial fluid and amniotic fluid) [17]. Their presence has also been demonstrated in the placenta [4], breast milk and infant meconium, indicating their potential impact on pregnancy outcomes and infant exposure [4,17]. Once inside the body, MNPs may cross biological barriers such as the placental barrier, the blood–brain barrier and the blood–testis barrier, which raises serious concerns [17].

Consumption of MNPs along with contaminated food may pose a health risk by causing or contributing to the development of various diseases. Studies suggest that exposure to MNPs may be associated with immunotoxicity, cancer, gastrointestinal, respiratory and cardiovascular diseases, as well as adverse pregnancy outcomes [4,17]. Additionally, MNPs can disrupt endocrine function, affecting organs such as the thyroid, testes and ovaries [12]. MNPs can also affect liver function [13,18,19].

### 1.7. MNPs in the Liver

The liver is a major organ exposed to xenobiotics, including MNPs, which can affect its function. MPs can damage the liver through the production of reactive oxygen species (ROS), contributing directly or indirectly to the development of steatohepatitis and increased fibrosis [13]. Mechanisms include oxidative stress, metabolic dysfunction and inflammatory response. MNPs can damage mitochondria, leading to disruption of key metabolic pathways and cycles such as the Krebs cycle, glycolysis or gluconeogenesis [19]. In addition, MNPs can induce apoptosis in hepatocytes [19]. The severity of MNP-induced liver damage is influenced by factors such as exposure duration, smaller particle size and early developmental stages [18].

### 1.8. Literature Selection Strategy

This narrative review was based on literature searches performed primarily in PubMed, supplemented by external web-based searches and manual screening of reference lists from key articles. The search included publications available up to the final preparation of the manuscript, with priority given to studies from 2020 to 2026, particularly the most recent evidence from 2024 to 2026. Older publications were included when they provided essential background on definitions, analytical methods, biological barriers, hepatic physiology, or established mechanistic concepts. Search terms included combinations of: “microplastics”, “nanoplastics”, “micro- and nanoplastics”, “MNPs”, “human exposure”, “gastrointestinal uptake”, “respiratory exposure”, “dermal exposure”, “iatrogenic exposure”, “translocation”, “blood”, “liver”, “hepatic accumulation”, “hepatotoxicity”, “oxidative stress”, “mitochondrial dysfunction”, “endoplasmic reticulum stress”, “inflammation”, “lipid metabolism”, “steatosis”, “fibrosis”, “FTIR”, “Raman spectroscopy”, “LD-IR”, “Py-GC-MS”, “AFM-IR”, “O-PTIR”, “DLS” and “NTA”. Studies were selected based on their relevance to MNP exposure, systemic distribution, analytical detection and mechanisms of liver injury, with emphasis on recent and human-relevant evidence. As this was a narrative review, article selection was guided by thematic relevance rather than by a formal systematic review protocol or meta-analysis.

### 1.9. Scope and Novelty of the Review

The aim of this study is to summarize the current knowledge regarding the routes of exposure to MNPs, the mechanisms of their entry into the body, their transport in the circulation, and their accumulation in the liver. This review integrates these topics into a single framework, illustrating the successive stages from human exposure to MNPs to their potential role in liver damage. This approach distinguishes this study from many previous works, in which exposure, distribution, detection, and hepatotoxicity were discussed separately. Particular attention is also devoted to methods for detecting MNPs in biological material, including in vivo and postmortem studies, as well as the limitations of these methods, as they are of great importance for interpreting data on particle accumulation in tissues. The study also focuses on the molecular mechanisms of MNP hepatotoxicity, their potential impact on liver function, and the limitations of current experimental research.

## 2. Entry of Micro- and Nanoplastics into the Human Body

MNPs, initially regarded mainly as a threat to the environment and biodiversity, are increasingly being recognized as a potential risk to the health of living organisms, including humans, due to their ability to enter the body and bioaccumulate [20,21]. In Section 2, we focus primarily on the routes by which MNPs may enter the human body, whereas Section 3 discusses their fate after entering the organism. The main routes by which MNPs may enter the human body are: (i) the gastrointestinal tract, (ii) the respiratory system and (iii) the skin [20,22]. However, these routes should not be considered equivalent in terms of systemic uptake. Current evidence supports gastrointestinal and respiratory pathways more strongly, whereas dermal uptake through intact skin appears to be a lower-probability and less substantiated route [20,22,23,24].

Importantly, the exposure routes discussed below differ substantially in the strength of supporting evidence. Gastrointestinal exposure is currently supported by the broadest body of evidence, including dietary exposure data, intestinal barrier models, and mechanistic studies describing mucus, glycocalyx, epithelial, and junctional filters, as well as potential access to vascular or lymphatic compartments [20,22,25,26,27,28,29,30,31,32,33,34,35,36,37,38,39,40,41,42,43,44,45,46,47,48,49]. The respiratory route is also biologically plausible and supported by deposition, epithelial uptake, and extrapulmonary distribution studies, although direct human evidence for efficient alveolar-to-blood translocation remains limited [50,51,52,53,54,55,56,57,58,59]. In contrast, dermal uptake should be interpreted with greater caution, as available evidence mainly demonstrates local penetration in ex vivo or reconstructed skin models rather than confirmed systemic entry in humans [23,24,60,61]. Iatrogenic exposure may bypass epithelial barriers and therefore represents a plausible direct entry route; however, current data mainly demonstrate particle release from medical materials rather than the actual quantitative burden, retention, or clinical consequences in patients [62,63,64,65,66,67]. This relative hierarchy of evidence is summarized in Table 1.

### 2.1. Gastrointestinal Uptake of MNPs

The gastrointestinal tract, particularly the intestine, is considered one of the main routes of exposure and potential entry of MNPs into the human body [20,22]. Because direct human evidence for intestinal translocation of MNPs remains limited, part of the current mechanistic understanding is extrapolated from studies on food-grade particles, model nanoparticles and general intestinal barrier physiology. For conceptual clarity, intestinal barrier crossing can be viewed as passage through four sequential filters: (i) the mucus layer, (ii) the glycocalyx and brush border, (iii) the epithelial cell layer involved in transcellular uptake and (iv) the intercellular junctional complex relevant to paracellular passage. Once these barriers are crossed, MNPs enter the lamina propria, where their further interaction with immune cells and access to blood capillaries or lymphatic vessels may determine their systemic distribution [25,26,27,28,29].

The first filter is the mucus layer, a viscoelastic network composed of mucins, lipids, proteins, exfoliated cells and bacterial components. In the intestine, this layer is produced primarily by goblet cells and is based largely on the MUC2 mucin scaffold, while also containing secretory immunoglobulin A (sIgA) and antimicrobial peptides [25,26,27]. Studies on particle transport through native intestinal mucus have shown that nanoparticle mobility depends strongly on surface charge, particle size and the surrounding food matrix; notably, anionic particles moved through mucus more rapidly than cationic particles, whereas in the presence of lipids, increasing particle size from 100 to 500 nm markedly reduced diffusion [30]. In a more recent cellular model incorporating a mucus layer, 50 nm polystyrene particles were still able to reach the basolateral side, but substantially less efficiently than in simpler models lacking a more complete mucus barrier [31]. Thus, adhesion to mucins may trap MNPs and facilitate their removal with luminal contents, whereas smaller particles with surface properties less prone to mucoadhesion are more likely to reach the epithelial surface [31,32]. Importantly, in vitro gastrointestinal digestion itself may modify particle surface properties and protein corona composition, thereby markedly increasing transepithelial translocation of selected polystyrene nanoparticles in Caco-2/HT29-MTX co-culture models [33]. 

The second filter is formed by the glycocalyx and brush border, which separate the mucus layer from the apical membrane of epithelial cells. Classical work by Frey et al. demonstrated that even a relatively thin glycocalyx can effectively restrict the access of larger particles to epithelial membrane receptors: 28.8 nm CTB-gold probes bound to M cells but not to enterocytes, whereas 1.13 μm particles did not efficiently bind to mature enterocytes [34]. The authors further estimated that a glycocalyx thickness of approximately 20 nm was sufficient to prevent 1 μm microparticles from reaching the cell surface [34]. This helps explain why microfold cells (M cells) are considered particularly important portals for particle uptake. Because M cells display a reduced glycocalyx and less developed microvilli than conventional enterocytes, they provide a more permissive interface for the uptake of larger particles, including MNPs [35,36].

The third filter is the epithelial cell itself, most importantly the enterocyte or the M cell. At this stage, MNPs must cross the cellular barrier through passive or active processes. For very small nanoplastics, passive diffusion may contribute to uptake, but endocytosis and transcytosis are generally thought to play the dominant role. This interpretation is supported by a recent mechanistic tri-culture study showing that inhibition of ATP synthesis reduced 26 nm carboxylated polystyrene translocation by only 35%, consistent with a mixed contribution of passive diffusion and active uptake pathways including phagocytosis and clathrin-related endocytosis [37]. In a human duodenum-chip model, 25 nm polystyrene MNPs were taken up through both passive and active mechanisms dependent on actin and dynamin [38]. In an improved human follicle-associated epithelium model containing M cells, the transport of 200 nm carboxylated polystyrene particles was greater across FAE/M-cell-type epithelium and its inhibition by EIPA and nystatin suggested the involvement of macropinocytosis-related pathways [39]. Particle size appears to be a major determinant at this step. This was also supported by Walczak et al. [40], who showed in intestinal cell models of increasing complexity that translocation was strongly size- and surface-dependent, reaching up to 7.8% for 50 nm polystyrene nanoparticles but only 0.8% for 100 nm particles, while mucus and M-cell incorporation substantially modified absolute transport rates. In human intestinal organoid-derived epithelial models, particle internalization depended on size, time and concentration and M cells were particularly efficient in capturing larger particles [36]. In mice, even 1 and 10 μm polystyrene microplastics were visualized crossing the intestinal mucus and epithelial barrier, while 1 μm particles were subsequently detected in plasma [41]. Quantitative support for this size dependence was provided by Choi et al. [42], who reported after 72 h approximately 27.3% translocation for 50 nm particles, 45.1% for 100 nm particles, and 10.2% for 500 nm particles across Caco-2 monolayers, while M-cell incorporation preferentially enhanced the passage of larger 500 nm particles. Together, these findings indicate that the cellular barrier is effective, but not absolute, especially for smaller particles, higher doses, or prolonged exposure.

The fourth filter is the intercellular junctional complex, particularly tight junctions, which regulate the paracellular route. Under physiological conditions, this pathway is tightly restricted; however, it may still contribute to early particle uptake. In the study by Coméra et al., performed with food-grade TiO_2_ particles, blocking tight-junction permeability reduced villus uptake by 66%, and the authors concluded that passive diffusion through paracellular spaces represents one of the major early uptake routes [43]. Although this work did not investigate MNPs directly, it provides important mechanistic support for the idea that not all particles must necessarily be internalized by epithelial cells in order to cross the intestinal barrier. This route may be particularly relevant under inflammatory conditions, when intestinal permeability is increased [43,44].

After crossing the epithelial barrier, MNPs enter the lamina propria rather than the bloodstream directly. At this stage, their further fate may depend on interactions with immune cells and their access to vascular or lymphatic compartments. Immune cells, especially dendritic cells and lymphocyte-rich structures associated with Peyer’s patches, may sample luminal or translocated material and contribute to its local sequestration or downstream immune handling [45,46]. If particles cross through ordinary villi, they may subsequently access villus capillaries and thereby enter the portal circulation [38,43]. Alternatively, lymphatic transport may also contribute, as lacteals are structurally specialized for the uptake of interstitial fluid, macromolecules and lipids and may provide a route for further transport of particles that have already crossed the intestinal epithelium [47]. Older studies using orally administered latex particles demonstrated translocation of particles to mesenteric lymphatic structures and lymph nodes [48], while more recent nanoparticle-based oral delivery studies have shown that chylomicron-associated transport may facilitate movement from enterocytes into lymph and ultimately into the systemic circulation through the thoracic duct [49].

In summary, gastrointestinal translocation of MNPs should be understood not as a single event of epithelial crossing, but as a cascade of sequential barrier interactions followed by access to vascular or lymphatic compartments. Although both portal and lymphatic routes appear biologically plausible, the efficiency of this process remains low and is influenced by particle size, surface chemistry and intestinal barrier status [3].

### 2.2. Respiratory System

The respiratory system is another key route in the pathophysiology of MNP entry into the human body [1]. At the same time, it should be emphasized that this route has been characterized much better in animal and in vitro models than in humans [50,51,52,53].

Before a particle can reach the epithelium, it must first deposit in an appropriate region of the respiratory tract. Larger particles tend to be retained in the nose, pharynx, and bronchi, whereas smaller particles are more likely to reach distal regions of the lung. For this reason, small nanoplastics reaching the alveoli are considered more plausible candidates for translocation into the bloodstream [54]. This is indirectly supported by the study of Zhang et al. [53], in which, after inhalation exposure, 20 nm polystyrene (PS20) was cleared from the lungs and distributed to extrapulmonary tissues more efficiently than 100 nm polystyrene (PS100), and by the PET study of Shanmugiah et al. [52], in which 20 nm nanopolystyrene (nPS) left the lungs more rapidly and showed greater distribution to other organs than 0.2–0.3 μm micropolystyrene (mPS).

In the nose, trachea and bronchi, the main barrier is mucociliary clearance (MCC). The epithelium is covered by the airway surface layer, which consists of two components: the mucus layer and the periciliary layer (PCL). Cilia propel mucus toward the pharynx, while particles trapped within it are either expectorated or swallowed [55]. This appears to be one of the principal mechanisms limiting the further passage of MNPs and may also explain why some inhaled particles eventually end up in the gastrointestinal tract [53]. This upper-airway barrier is not only physical but also cellular, as polystyrene MNPs have been shown to be internalized by human nasal epithelial cells and to induce local toxicity after intranasal exposure in rats [56].

If a particle bypasses bronchial mucus and reaches the distal lung, it encounters a thin layer of alveolar lining fluid and surfactant. The alveolar–capillary barrier has three main layers: (i) the alveolar epithelium, (ii) the extracellular matrix/basement membrane, and (iii) the capillary endothelium. The epithelium is covered by a thin aqueous surfactant layer, which reduces surface tension but also modifies particle behavior [57]. Thorley et al. [51] demonstrated that surfactant proteins bind nanoparticles and that particle functionalization increases their interaction with surfactant and may enhance epithelial uptake. This indicates that surfactant may either hinder or facilitate subsequent cellular internalization.

Another important filter is formed by alveolar macrophages, which effectively remove micrometer-sized foreign bodies. However, Thorley et al. [51] also emphasized that smaller, individual nanoparticles may not be recognized as efficiently by macrophages and may instead be internalized directly by the alveolar epithelium. This again suggests that the smaller the particle, the greater the chance of bypassing this filter and proceeding directly to the epithelial stage. This may explain why microplastics are more often considered in the context of retention and local inflammation, whereas nanoplastics are more frequently discussed in relation to systemic translocation. This does not mean that microplastics cannot leave the lung at all, but biologically NPs appear to have a clearer advantage. This is supported by epithelial model studies by Yacobi et al. and Thorley et al., as well as by biodistribution studies by Shanmugiah et al. and Zhang et al., in which smaller particles showed more pronounced extrapulmonary distribution [50,51,52,53].

The next checkpoint is the alveolar epithelium, which is composed mainly of thin squamous alveolar type I (ATI) cells and cuboidal alveolar type II (ATII) cells. Thorley et al. [51] showed that ATI cells are the main determinants of nanoparticle uptake and translocation, as they internalized nanoparticles, whereas primary ATII cells showed little uptake. At the same time, no passage through tight junctions was demonstrated, indicating that the paracellular route was unlikely in the healthy model. For 50 nm particles, non-endocytic or diffusion-like processes appeared to contribute substantially, whereas for 100 nm particles clathrin- and caveolin-mediated endocytosis predominated.

After crossing the alveolar epithelial cell, the particle is still not yet in the bloodstream. It must also pass through the ultrathin ECM/basement membrane and the interstitial space. Classical physiology of the blood–gas barrier describes three layers—epithelium, ECM and endothelium—with type IV collagen playing a central structural role in the ECM. Although this layer may be extremely thin, it still constitutes a real selective barrier [57].

Only after crossing the capillary endothelium can the particle enter the bloodstream. This stage is difficult to observe directly under the microscope; therefore, it is usually inferred from the appearance of particles in blood and extrapulmonary organs. The strongest argument here comes from the study by Zhang et al. [53], in which, after respiratory exposure, blood concentrations of PS were low but detectable, while significant accumulation in the liver was also observed, which the authors interpreted as evidence of crossing the blood–air barriers. In the PET study by Shanmugiah et al. [52], nPS was likewise detected in multiple extrapulmonary organs after intratracheal administration. Additional in vivo support comes from intratracheal exposure studies with polyethylene microplastics, in which particles were detected predominantly in the lung but also in extrapulmonary tissues such as the liver and intestine, supporting at least limited post-pulmonary distribution [58]. At the same time, not all in vitro lung models indicate efficient barrier crossing, as recent bronchial and alveolar epithelial systems have shown particle internalization without overt barrier disruption or detectable translocation to the basolateral compartment [59].

### 2.3. Skin

Dermal exposure to MNPs may occur through direct contact with personal care products, cosmetics, sunscreens, facial cleansers, hand soaps, synthetic textiles, household dust and contaminated occupational or domestic surfaces [14]. Compared with the gastrointestinal and respiratory routes, dermal uptake currently appears to be the least substantiated pathway for the systemic entry of MNPs [20]. In the study by Döge et al. [23], performed on full-thickness human skin, fluorescently labeled polystyrene nanoparticles of 20 and 200 nm accumulated mainly in the stratum corneum and in the upper parts of hair follicles, whereas deeper penetration was observed only as a rare event at sites of focal particle aggregation. This argues against easy passage across an intact skin barrier into the circulation. Similar conclusions were reported by Zou et al. [24], who showed in excised human skin that hydrolyzed polystyrene nanoparticles of 25, 50 and 100 nm localized in the stratum corneum and hair follicles but did not penetrate the viable epidermis or dermis; even barrier disruption by tape stripping did not induce deeper penetration, whereas more evident penetration was observed only when dimethyl sulfoxide (DMSO) was used as a vehicle.

A different picture was obtained by Martin et al. [60], although it should be emphasized that their work was based on models with an impaired skin barrier, including ex vivo human skin after removal of the stratum corneum. Under these conditions, 100 nm and 500 nm nanoplastics were taken up by keratinocytes and fibroblasts, with 100 nm particles penetrating more efficiently than 500 nm particles. After removal of the stratum corneum, the particle signal in the epidermis increased from 9.23 ± 2.70 to 38.87 ± 7.52 pixels/cell, indicating that barrier damage may substantially increase the risk of nanoplastic entry into viable skin layers.

The strongest signal supporting the possibility of deeper penetration comes from the study by Song et al. [61], in which fragmented polystyrene penetrated into the dermal layer within 1 h in a 3D human skin model and for particles smaller than 2 μm the mean maximal penetrated amount reached 4.7 μg. The authors also confirmed penetration in an ex vivo model of human abdominal skin. However, it should be stated clearly that this study documented penetration into skin rather than direct measurement of particle concentrations in blood. Therefore, evidence for skin-to-blood passage in humans remains indirect rather than direct. 

Importantly, most available evidence is derived from ex vivo human skin, reconstructed 3D skin models, and fluorescently labeled polystyrene particles, which limits direct extrapolation to real-world dermal exposure in humans.

Skin barrier damage may increase local MNP penetration, as shown in impaired skin models and after removal of the stratum corneum [60,61]. Therefore, wounds and wound-care materials, including adhesive dressings, may represent a plausible scenario of increased local exposure; however, direct evidence that such products cause clinically relevant systemic MNP entry remains insufficient [23,24,60,61].

### 2.4. Iatrogenic and Direct Bloodstream Exposure

In addition to environmental exposure routes, medical procedures may represent a distinct iatrogenic pathway of MNP entry into the human body. Unlike ingestion, inhalation, or dermal contact, intravenous infusions and injections may bypass epithelial barriers and introduce particles directly into the bloodstream [62,63,64,65,66,67]. MNPs have been reported in intravenous infusion products, hypertonic fluids, disposable PVC infusion tubes, infusion bags, infusion sets and blood collection needles [62,63,64,65,66,67]. For example, hypertonic fluids contained an average of 62.82 ± 72.38 MPs/1000 mL, whereas disposable infusion tubes and blood collection needles released numerous particles, many within the 10–30 μm range [64,67]. These findings suggest that infusion systems, injections, catheters and other plastic medical materials may constitute a direct but still insufficiently characterized source of internal MNP exposure, particularly in patients requiring repeated or prolonged intravenous therapy. However, the quantitative contribution of this route to the total human MNP burden and its clinical significance remain uncertain [62,63,64,65,66,67].

## 3. Post-Entry Fate, Detection and Postmortem Identification of Micro- and Nanoplastics

MNPs have been found in various organs, including the kidneys [68], brain [68], lungs [68], placenta [69] and liver [68]. This suggests that these particles can spread throughout the body after overcoming biological barriers. Because organ deposition ultimately depends on vascular access, the liver, a key target for these particles in circulation, is especially important [68].

### 3.1. Systemic Translocation, Bloodborne Transport and Hepatic Sequestration of MNPs

After crossing the epithelial barrier, MNPs may first enter the subepithelial tissues, where they interact with local immune cells, including macrophages and dendritic cells. At this stage, they may be retained locally, trigger inflammatory responses, undergo phagocytosis, or be transported to lymphatic structures together with migrating immune cells [70]. However, only a fraction of MNPs subsequently move beyond the local subepithelial compartment and gain access to lymphatic or vascular pathways. Experimental models of the intestine indicate that this step appears to be influenced by particle size. In a human in vitro intestinal epithelial model, particles with a diameter of 100 nm exhibited greater permeability across the barrier than particles of 50 and 500 nm. This supports the idea that smaller fractions might have a greater potential for further systemic distribution [42,71].

Because of their large surface area, hydrophobic properties and the presence of functional groups, MNPs can adsorb a wide range of chemical and biological substances, forming a so-called ecocorona. This adsorbed layer may increase particle uptake by cells, modify interactions with epithelial and immune cells and potentially promote further translocation into the bloodstream. At the same time, compounds released from the plastic itself, as well as toxic substances bound to its surface, may contribute to pro-inflammatory, oxidative and cytotoxic effects. As a result, the toxicity of microplastics may depend not only on the particle itself, but also on its ability to carry other harmful agents [72,73].

After entering the bloodstream, the distribution of MNPs originating from the gastrointestinal tract is expected to be strongly influenced by the portal venous system, which transports blood from the intestines directly to the liver. The liver may represent an early and important filtration site for blood draining from the gastrointestinal tract and is therefore particularly exposed to particulate material that has crossed the intestinal barrier. Consequently, the liver may represent one of the early sites of accumulation or biological interaction for MNPs following oral exposure. Kupffer cells, the resident macrophages of the hepatic sinusoids, play a key role in their uptake owing to their ability to phagocytose and sequester foreign material circulating in the blood. The extensive sinusoidal network, the relatively slower blood flow within this area and the specialized hepatic phagocytic system all favor the retention of particles transported from the gut. This supports the view that the liver may be an important site of early accumulation and subsequent biological interactions [74,75,76]. Furthermore, experimental studies indicate that Kupffer cells may represent a functionally important target of microplastics in the liver. Exposure to polystyrene microplastics enhanced pyroptosis and inflammatory responses in these cells, implying that the interaction of MNPs with hepatic sinusoidal macrophages may contribute to acute liver injury [77].

A study by Li et al. demonstrated that oral administration of polystyrene nanoparticles (PSNPs) resulted in their very rapid distribution to the liver, detectable as early as 10 min after exposure, with maximal accumulation reached after 3 h. Quantitative analysis using pyrolysis–gas chromatography–mass spectrometry (Py-GC-MS) showed that, after 3 h, as much as 59.4% of the total administered PSNP dose could be present in the liver. The rapid appearance of PSNPs in the liver after intragastric administration is consistent with the possibility that, once the intestinal barrier is crossed, these particles may quickly enter the vascular compartment and be transported to the liver via the portal circulation [78].

In the case of inhalational exposure, MNPs that reach the alveolar region and cross the alveolar–capillary barrier may potentially gain access to the pulmonary capillaries and subsequently enter the systemic circulation via the pulmonary veins and the left side of the heart. Although direct evidence for this pathway in relation to MNPs in humans remains limited, studies on other inhaled particulate materials indicate that extrapulmonary redistribution to organs such as the liver, kidneys and spleen may occur following pulmonary deposition. At the same time, a portion of the material may be cleared from the lungs through mucociliary transport and, after swallowing, secondarily enter the gastrointestinal tract [79,80].

Taken together, these data indicate that once MNPs gain access to the circulation, they may show a tendency toward early sequestration in the liver, which, owing to its vascular architecture and abundance of phagocytic cells, constitutes one of the principal sites of their biological interaction. The significance of these interactions for the development of liver injury and its associated pathological mechanisms will be discussed in greater detail in Section 4.

### 3.2. Analytical Detection of Micro- and Nanoplastics in Biological Tissues: Methods and Methodological Challenges

Reliable detection of MNPs in biological tissues remains methodologically challenging. These particles must first be separated from complex organic matrices containing proteins, lipids and cellular debris. This difficulty is further compounded by the very low particle-to-tissue mass ratio, even in organs in which MNP accumulation has been demonstrated, as well as by the risk of analytical artifacts caused by contamination, non-plastic particulate matter and matrix-related interference. An additional and particularly important limitation is particle size itself: whereas larger microplastics can often be detected using established spectroscopic procedures, reliable identification of nanoplastics remains substantially more difficult because current analytical platforms lose sensitivity and resolution at the submicron scale. As a result, the smallest and potentially most bioavailable fraction of the plastic burden may be systematically underestimated [81]. Current detection strategies therefore rely on multistep workflows combining sample pretreatment with vibrational spectroscopy, thermal analysis and microscopy-based methods, most commonly Fourier transform infrared spectroscopy (FTIR), laser infrared spectroscopy (LD-IR), Raman spectroscopy, Py-GC-MS, complementary imaging techniques and newer advanced approaches for nanoplastic detection [72,82,83,84,85].

It is also important to note that the analytical techniques used for MNP detection differ not only in sensitivity and spatial resolution, but also in their qualitative and quantitative capabilities. Some methods are better for qualitative particle identification, whereas others are more useful for particle counting, size analysis, or measuring the amount of polymer in biological tissues [86].

Moreover, detection of MNPs in human tissues should not be interpreted as direct evidence of causality. Current human data mainly support the plausibility of exposure, systemic distribution and organ-level accumulation, whereas causal inference regarding liver injury relies primarily on experimental models [68,83,87,88].

#### 3.2.1. Sample Pretreatment

Pretreatment of biological samples for the detection of micro- and nanoplastics primarily involves the removal of the complex organic matrix, such as proteins, lipids and cellular debris, while preserving particle integrity. To this end, chemical or enzymatic digestion is used, most commonly with reagents such as KOH, H_2_O_2_, Fenton’s reagent, HNO_3_, or proteinase K, followed by density separation using high-density solutions such as ZnCl_2_ or NaI. Subsequent steps usually include centrifugation, filtration and concentration of the material on filters compatible with downstream spectroscopic analysis. This step is of critical methodological importance, because both insufficient sample purification and overly aggressive digestion may lead to analytical interference, particle loss, or damage to certain polymers [72,89]. Only after these steps have been completed can reliable application of downstream particle identification methods—primarily vibrational spectroscopy, thermal analysis and imaging techniques, discussed in the following subsections—be achieved.

#### 3.2.2. Spectroscopic Methods for MPs

Spectroscopic techniques, particularly FTIR, LD-IR and Raman spectroscopy, are widely used in the analysis of micro- and nanoplastics, as they enable polymer identification on the basis of characteristic spectral signatures [90].

FTIR, especially in its microspectroscopic variant (µFTIR), combines microscopic visualization of individual particles with infrared spectral analysis. Polymer identification is then performed by comparison of the obtained spectra with reference libraries. In biological samples, analysis is usually preceded by digestion, filtration and deposition of the material on a substrate compatible with infrared radiation, which also makes it possible to assess the chemical composition, size and morphology of the particles [91]. FTIR/µFTIR is primarily a qualitative tool for polymer identification, while in imaging-based workflows it also enables semi-quantitative assessment of particle counts, size distribution and relative polymer abundance. However, its usefulness for accurately measuring large amounts of mass is still not as good as that of pyrolysis-based methods [86].

In practice, the detection limit for particles analyzed by FTIR is usually around 10 µm, whereas µFTIR may enable identification of even smaller microplastic particles, sometimes in the range of approximately 6–20 µm [92]. The main advantages of this method include relatively rapid chemical identification of solid samples and some liquid samples, limited requirements for sample preparation, good reproducibility, relatively low cost and the ability to generate high-quality spectra characteristic of specific polymers. Its limitations include interference from background signals and sample matrix effects, as well as limited capacity to detect particles within the nanometer range [92].

FTIR/µFTIR enables the detection of microplastics in biological samples, including whole blood [91], placenta [93], stool [94], urine [95] and sputum [96]. In addition, studies have reported the use of this method for MP detection in the lungs [97], colon [98], and synovial tissue [99]. Leonard et al. demonstrated the presence of microplastics in whole blood in 18 of 20 participants (90%), with plastic concentrations ranging from 1.84 to 4.65 μg/mL. This observation is of considerable biological relevance, as it confirms that MPs may be transported via the bloodstream and may therefore potentially reach filtering and metabolically active organs such as the liver [91]. Furthermore, analysis of human saphenous vein samples identified microplastic particles characterized by substantial size variability, with a mean length of 119.59 ± 226.82 µm and a mean width of 41.27 ± 62.80 µm. These findings indicate that FTIR/µFTIR techniques enable the identification of particles across a broad size range in human vascular tissue, encompassing both smaller MP fractions and distinctly larger particles. This may suggest the potential applicability of FTIR/µFTIR to the identification of similarly sized particles in other types of human tissues [100]. Furthermore, MPs have been identified and characterized in human placental tissue using different spectroscopic and analytical approaches [83,101].

Another important method is Raman spectroscopy. This method offers a valuable addition to infrared techniques for studying microplastics in biological samples, particularly when the goal is to identify smaller particles within complex tissue environments. Raman spectroscopy is primarily a qualitative tool. It may provide semi-quantitative data on particle counts and size distribution. However, its broader quantitative application is limited by low throughput and longer analysis time compared with FTIR-based workflows [102].

Its application has been described, among others, in studies of human placenta [103], breast milk [104], semen [105], stool [106], urine [107], lung tissue [108], liver [87], spleen [87] and kidney [87], although the method remains limited by background fluorescence and the high complexity of biological matrices.

In a study using confocal Raman microspectroscopy with an inVia™ confocal Raman microscope (Renishaw plc, Wotton-under-Edge, United Kingdom), placentas from 50 women after delivery were analyzed, and 40 microplastic particles were identified in 31 of the 50 samples. The mean size of the detected particles was 2.35 ± 1.25 µm, with a range of 1.03–6.84 µm. These findings suggest that confocal Raman microspectroscopy may be particularly useful for detecting smaller microplastic particles than FTIR-based techniques, owing to its higher spatial resolution and lower detection threshold [103].

#### 3.2.3. Thermal Method

In the realm of thermal methodologies employed in MP analysis, Py-GC-MS distinguishes itself by focusing on the identification of polymer pyrolysis products rather than the particles themselves. The main advantages of this technique include high analytical sensitivity and specificity. Furthermore, its operational flexibility is enchanced by the capacity to function in both full-scan mode and selected ion monitoring (SIM), thereby adapting to the specific analytical objectives at hand. Py-GC-MS enables qualitative polymer identification and provides strong quantitative capability for determining total polymer mass or concentration. However, because it is not a particle-resolved technique, it does not provide information on the number, shape, size, or localization of individual particles [90].

A particular challenge is posed by endogenous organic compounds such as lipids, which may generate degradation products similar to those observed for certain polymers, especially PE, leading to false-positive results. In addition, some plastics, such as PVC, yield relatively nonspecific pyrolysis products, making unequivocal identification more difficult. Therefore, the effectiveness of Py-GC-MS in biological studies depends heavily on careful sample preparation and rigorous quality-control procedures [109,110].

Available studies indicate that Py-GC-MS may be a useful method for the determination of MPs in human blood [110], testes [111] and semen [111]. Nardella et al. emphasized that this method allows quantitative polymer analysis in biological samples, and recent studies using Py-GC-MS have reported the presence of polymers in blood, arteries, bone marrow and placenta, with concentrations varying substantially between studies—from approximately 1.1 µg/mL in blood to approximately 126.8 µg/g in placenta. At the same time, the authors noted that although Py-GC-MS offers a major advantage in bulk polymer quantification, it is limited by the indirect nature of the analysis and the risk of nonspecific signals, especially in complex biological matrices. For this reason, the method should be regarded primarily as a complementary tool rather than a direct substitute for particle-based analytical approaches [110].

#### 3.2.4. Microscopy and Imaging Techniques

Microscopy and imaging techniques, such as scanning electron microscopy (SEM), transmission electron microscopy (TEM), atomic force microscopy (AFM), confocal microscopy and fluorescence-based approaches using Nile Red, serve mainly as complementary tools in MP analysis [72]. Fluorescence microscopy is a simple and quick way to screen samples in Nile Red-based workflows because it makes it easy to see stained plastic-suspect particles. Its primary utility resides in initial observation and semi-quantitative evaluation, while spectroscopic validation may still be necessary within the analytical process [112,113].

Microscopy- and imaging-based methods are primarily qualitative tools and enable assessment of particle morphology, topography, localization and, in some cases, cellular internalization. Their quantitative value is usually limited to determining particle size and counting particles within the field of view; however, in most cases they do not independently provide unequivocal chemical identification of polymers and are therefore most often combined with spectroscopic methods. These techniques are particularly useful for the analysis of small particles and for visualizing their presence in complex biological samples, although fluorescence-based methods such as Nile Red require cautious interpretation because of the possibility of staining residual organic matter and generating false-positive results [114,115,116].

In practice, conventional light and stereomicroscopy remain useful mainly for the analysis of larger particles, with practical resolution generally above 1 µm. Higher imaging capability is provided by SEM and TEM, with SEM being particularly useful for evaluating particle morphology and surface structure, whereas TEM allows the analysis of even smaller structures, including submicron and nanometric fractions. AFM enables very high-resolution imaging of surface topography; however, its use in studies of microplastics in human cells remains limited to date [72,115]. In the case of fluorescence-based techniques using Nile Red, reported numerical values depend strongly on the entire analytical workflow. In some approaches, the practical limit for reliable manual counting and confirmation of particles was approximately 20–30 µm. In other studies, particles in the range of 50–1200 µm were analyzed, indicating that Nile Red is most valuable primarily as a screening tool for microplastics rather than as a definitive method for identifying the smallest particles [116,117].

#### 3.2.5. Advanced Techniques for MNP Detection

In the context of increasing human exposure to MNPs, methods capable of detecting very small particles are becoming particularly important. Conventional techniques such as FTIR and Raman spectroscopy are limited mainly to microplastics; therefore, more advanced approaches are increasingly being used in nanoplastic analysis, including atomic force microscopy–infrared spectroscopy (AFM-IR), optical photothermal infrared spectroscopy (O-PTIR), nanoparticle tracking analysis (NTA) and dynamic light scattering (DLS) [84,85].

AFM-IR and O-PTIR are advanced infrared techniques based on the photothermal effect and are designed for the analysis of very small MNPs. O-PTIR enables submicron-scale analysis, whereas AFM-IR achieves a resolution of approximately 20 nm, which makes it applicable to nanoplastic research. These methods extend the capabilities of conventional FTIR and Raman spectroscopy but still remain largely specialized tools; O-PTIR has already begun to be described in studies of human tissues, whereas AFM-IR remains primarily an experimental tool [84,118].

DLS and NTA, in turn, are promising methods for nanoplastic analysis because they allow assessment of particle size distribution and particle number in suspension, including in the sub-20 nm range. However, they do not provide information on particle chemical composition and in complex biological samples their results should therefore be interpreted with caution and regarded as complementary rather than stand-alone identification tools [85].

#### 3.2.6. Summary of Methods

A clear understanding of the analytical methods used for MNP detection is essential for interpreting the available data on their presence in the human body. Differences in sensitivity, spatial resolution and susceptibility to matrix interference among individual techniques may substantially affect study outcomes and their comparability. At the same time, the application of spectroscopic and analytical methods to biological samples such as blood and placenta provides direct evidence of the systemic bioavailability of microplastics and their ability to accumulate in tissues. In the context of portal circulation physiology and the filtering and immunological functions of the liver, this provides a rationale for further investigation of its potential role as a site of MNP accumulation [83,90,91,119]. Table 2 summarizes the main analytical methods used for MNP detection in biological samples, including their suitable sample types, approximate detection ranges, principal strengths and key limitations.

### 3.3. Ex Vivo and Postmortem Evidence of MNP Accumulation in Human Organs

Relevant evidence for the presence of MNPs in organs is provided by ex vivo analyses and postmortem studies, as these allow direct examination of solid tissues and assessment of true organ-level particle accumulation. In contrast to blood- or placenta-based studies, autopsy data enable a more direct evaluation of MNP distribution in specific organs, although they remain limited by small sample sizes and heterogeneity of the analytical methods used [120,121].

In an ex vivo study, Horvatits et al. demonstrated the presence of microplastics in human liver tissue, with apparently higher concentrations in samples obtained from patients with cirrhosis than in those from individuals without concomitant liver disease. Concentrations in the cirrhosis group ranged from 4.6 to 11.9 particles/g tissue, whereas in the control group they ranged from 0.3 to 1.9 particles/g and the difference between groups was statistically significant (*p* = 0.012). This observation suggests that chronically injured liver tissue may exhibit a greater tendency to retain microplastics, although the currently available data do not allow one to determine unequivocally whether this phenomenon is causal or secondary to the structural and hemodynamic changes associated with cirrhosis [87].

Nihart et al. reported the presence of MNPs in postmortem human liver, kidney and brain samples using complementary analytical techniques. In specimens collected in 2024, the median total plastic concentration reached 433 µg/g in the liver, whereas brain tissue showed a much higher burden (4917 µg/g), suggesting inter-organ differences in accumulation. Notably, concentrations in both the liver and brain were higher in 2024 than in 2016. Although the liver was not the most heavily burdened organ, its role in portal circulation, metabolism, and phagocytic clearance may make it a particularly important site of MNP retention in the context of systemic exposure [68].

In one of the most comprehensive postmortem studies to date, Dzierżyński et al. analyzed postmortem samples from the brain, liver, thyroid, kidneys, heart, lungs and skeletal muscle using advanced imaging and spectroscopic techniques. The highest levels were found in the thyroid (40.4 MP/g), brain (24.4 MP/g) and kidneys (21.5 MP/g), whereas the liver showed a moderate concentration (6.6 MP/g). Despite the lower concentration in the liver, the presence of MPs in this organ may be biologically important and once again supports the hypothesis outlined above [68,122]. Relatively high levels in the lungs may support a role for inhalational exposure, whereas the presence of MPs in the brain is consistent with the possibility that these particles may be capable of crossing biological barriers, including the blood–brain barrier. In the analyzed organs, detected microplastic particles were found mainly within the range of approximately 200–3000 µm, indicating that tissue retention may also involve larger particles, potentially trapped within the microcirculation or tissue structures [122].

Despite the growing number of studies, the currently available data remain fragmentary and precise determination of the level of MNP accumulation in human organs remains challenging owing to substantial methodological differences and the limited comparability of results [68,87,120,121,122].

## 4. MNPs and Molecular Changes Leading to Liver Disease

Available in vitro and in vivo evidence indicates that MNPs accumulate in the liver and disrupt key cellular processes, including redox homeostasis [88,123,124,125,126,127,128,129], mitochondrial function [6,124,130,131,132,133], induction of endoplasmic reticulum stress [134,135,136,137,138], inflammatory responses [88,123,129,134,139,140,141,142,143,144,145,146,147,148], de novo lipogenesis and lipid accumulation [127,129,137,149,150,151,152,153], as well as lipid catabolism [150,154,155,156,157,158]. In addition, MNPs may induce DNA damage [159,160,161,162,163]. These mechanisms may contribute to pathways associated with hepatocellular injury, fibrosis, and liver disease progression [147,164,165,166,167,168]. The magnitude of these effects depends, among other factors, on particle characteristics such as size, surface charge and the degree of environmental aging [6,133,138,155]. However, because most mechanistic data are derived from in vitro and animal models, the pathways discussed below should be interpreted as potential mechanisms of MNP-associated hepatotoxicity rather than definitive evidence of causal liver injury in humans.

**Figure 1:**
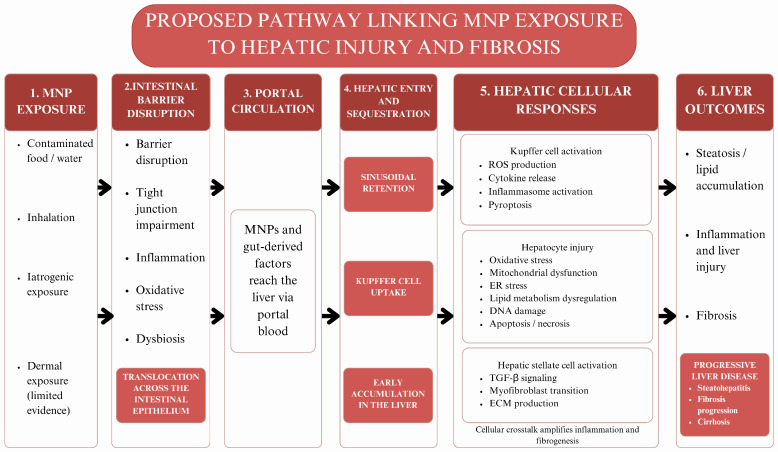
Proposed pathway linking MNP exposure to hepatic injury and fibrosis (figures were prepared using Canva Pro, https://www.canva.com, accessed 19 May 2026). MNPs may interact with the intestinal barrier, promote barrier disruption, inflammation, oxidative stress, and dysbiosis, and translocate across the intestinal epithelium. After entering portal circulation, MNPs and gut-derived inflammatory factors may reach the liver, where sinusoidal retention and Kupffer cell uptake may contribute to early hepatic accumulation. Subsequent Kupffer cell activation, hepatocyte injury, and hepatic stellate cell activation may drive inflammatory signaling, oxidative stress, mitochondrial dysfunction, ER stress, lipid dysregulation, DNA damage, ECM production, steatosis, and fibrosis.

At the same time, it should be noted that most studies evaluating the hepatic effects of MNPs focus on PS-MNPs, even though polystyrene accounts for only about 5% of global plastic production (Table 1 and Table 2). This may be due to the easy commercial availability of spherical, monodisperse and pristine PS particles. In contrast, PP-, PVC-, PU- and PET-MNPs are much more difficult to obtain as standardized test materials, which may constitute a major limiting factor in their use for assessing MNP-induced hepatotoxicity [169]. To facilitate interpretation of the mechanistic sections below, Figure 1 provides a schematic overview of the proposed pathway linking MNP exposure, intestinal barrier disruption, portal transport, hepatic sequestration, Kupffer cell activation, hepatocyte injury, hepatic stellate cell activation, and downstream liver outcomes, including steatosis, inflammation, and fibrosis.

### 4.1. Effects of MNPs on the Antioxidant System

It has been shown that MNPs can affect the antioxidant system, including the function of the Nrf2-Keap1 axis [88,123,124,125,126,127,128,129]. The Nrf2-Keap1 signaling pathway is one of the main mechanisms of cellular protection against oxidative stress; its activation involves the release of Nrf2 from its complex with Keap1, translocation of this factor into the cell nucleus and induction of transcription of genes encoding antioxidant and detoxifying enzymes that are involved in the maintenance of redox homeostasis [170].

Wen et al. showed that in mice and in AML-12 hepatocytes, PS-NPs can inhibit the Nrf2-dependent signaling pathway. This phenomenon was associated with excessive production of reactive oxygen species (ROS), increased oxidative stress in the liver and increased expression of pro-inflammatory factors such as NLRP3, IL-1β and caspase-1. At the same time, pharmacological activation of Nrf2 signaling was shown to alleviate oxidative stress and inflammation induced by PS-NPs [88]. Similar results were obtained in a study in albino rats, in which a decrease in the activity of antioxidant enzymes such as catalase (CAT), superoxide dismutase (SOD), glutathione peroxidase (GPx) and HO-1 (heme oxygenase-1) was observed after exposure to PS-NPs, with a concomitant increase in malondialdehyde (MDA), a marker of lipid peroxidation and an increase in ROS levels. These changes were associated with a decrease in Nrf2 expression [123].

On the other hand, a study in HepG2 and L02 cells showed Nrf2 activation after exposure to PS-NPs [125]. These discrepancies suggest that NPs may initially activate the Nrf2-Keap1 axis, but that this response may become exhausted over time. This hypothesis is supported by the results of Acar et al., in which an increase in the expression of genes encoding SOD and CAT was observed at lower concentrations of PS-NPs, while a decrease in the expression of genes encoding elements of the antioxidant system, such as GPx and glutathione S-transferase (GST), was observed at higher concentrations [126].

Similarly, a dose-dependent increase in the activity of antioxidant enzymes, such as GST, GPx and SOD, was observed after exposure to MPs in many studies, while others reported a decrease in the activity of antioxidant enzymes, a decrease in hepatic glutathione stores and an increase in ROS levels [124,127,128,129]. These phenomena may reflect dysfunction of the Nrf2-Keap1 axis, ranging from its initial protective activation to its subsequent depletion. However, it should be taken into account that discrepancies between study results may also be due to methodological differences (Table 3 and Table 4).

Furthermore, one study showed activation of the Nrf2 pathway after PS-MP exposure. However, this did not translate into increased expression of HO-1 (heme oxygenase-1) or NQO1 (NAD(P)H:quinone oxidoreductase 1), two enzymes involved in detoxification and antioxidant responses. This suggests an inefficient rather than effective antioxidant response. [174].

Another study using a co-culture model of HepG2 and THP-1 cells showed that polymethylmethacrylate (PMMA) MP, a widely used thermoplastic, affected HO-1 expression in a concentration-dependent manner: expression increased at higher concentrations but decreased at lower concentrations. In addition, a decrease in the level of the mitochondrial antioxidant enzyme SOD2 was observed at high PMMA-MP concentrations. These results suggest that PMMA-MPs may also activate the Nrf2-Keap1 axis and, at high concentrations, lead to its dysfunction [159]. MPs may also reduce the expression of the mitochondrial deacetylase sirtuin 3 (SIRT3), which plays an important role in maintaining SOD2 activity and regulating ROS levels [124].

Taken together, these data suggest that MNPs may disrupt hepatic antioxidant mechanisms, particularly by affecting the Nrf2-Keap1 axis and related pathways involved in redox homeostasis. These changes may contribute to ROS accumulation and oxidative stress. However, the direction of the response is not entirely clear and appears to depend largely on the tested dose.

### 4.2. MNP-Induced Mitochondrial Damage

Mitochondria play a key role in the energy metabolism of hepatocytes, including the oxidation of fatty acids and carbohydrates. Increasing evidence suggests that mitochondrial dysfunction is an important contributor to the pathogenesis of MAFLD and liver fibrosis [180].

Available data indicate that MNPs can disrupt mitochondrial structure and function [6,124,130,131,132,133]. Exposure to MNPs in mice has been shown to cause morphological changes in liver mitochondria, such as swelling and loss of mitochondrial cristae [124,131]. In addition, the decrease in OPA1 observed after exposure to PS-NPs reflects impaired mitochondrial fusion and disrupted cristae organization [130,181]. These changes are accompanied by a decrease in mitochondrial membrane potential (ΔΨm) and increased production of ROS, leading to oxidative stress and damage to cellular components [124,131,132,133]. Excessive mitochondrial ROS production can also initiate necroptosis in macrophages, indirectly contributing to hepatocyte damage [131].

Moreover MNPs may impair respiratory chain function and ATP synthesis, exacerbating cellular dysfunction [6,131]. For example, aged MPs, which predominate in the environment, have been shown to decrease the activity of respiratory chain complexes I and IV and induce reductive stress associated with an increase in the NADH/NAD^+^ ratio, which correlates with increased hepatotoxicity [6]. Disruption of mitochondrial dynamics also appears to be an important mechanism. In studies on human liver cells, exposure to PS-NPs was associated with increased expression of Drp1 and its phosphorylated form (p-Drp1), suggesting enhanced mitochondrial fragmentation linked to cell death [130]. In the same studies, decreased expression of PGC-1α, an important regulator of mitochondrial biogenesis and function, was observed, which may favor redox imbalance, exacerbate oxidative stress and exacerbate mitochondrial dysfunction. Impaired PGC-1α expression is also associated with the development of metabolic and inflammatory diseases, including hepatic steatosis [130,182].

Overall, MNPs can impair mitochondrial integrity and function in liver cells, inducing structural changes, respiratory chain dysfunction, increased oxidative stress and abnormalities in mitochondrial dynamics.

### 4.3. MNPs, Endoplasmic Reticulum Stress, Autophagy and Regulated Cell Death

Endoplasmic reticulum (ER) stress develops when ER homeostasis is disrupted, causing unfolded or misfolded proteins to accumulate and triggering the unfolded protein response (UPR). Although the UPR initially acts as a protective mechanism, persistent ER stress can eventually drive cell death. The liver seems to be particularly sensitive to this process, which has been implicated in the development of disorders such as NAFLD [183].

Studies suggest that ER stress may be one of the main mechanisms underlying the hepatotoxicity of MNPs [134,135,136,137,138]. PS-MNPs have been shown to activate the ER stress response involving the PKR-like ER kinase (PERK)–eukaryotic translation initiation factor 2α (EIF2α)–activating transcription factor 4 (ATF4) pathway, and activation of another protein in this pathway, C/EBP homologous protein (CHOP), has also been reported for PS-MPs [135,136,137]. Activation of this pathway in hepatocytes can lead to increased production of ROS, decreased ΔΨm, increased Parkin-dependent mitophagy and induction of apoptosis [135]. Mitophagy in the liver appears to have a complex role: on the one hand, it enables the clearance of damaged mitochondria and helps maintain metabolic homeostasis in hepatocytes; on the other hand, its excessive or impaired activation can exacerbate liver cell damage and promote the progression of liver diseases, including steatosis and fibrosis [180].

Activation of the PERK–ATF4 pathway may also increase TRIB3 protein expression, leading to inhibition of AKT/mTOR signaling and promotion of autophagy. This process is accompanied by changes in the expression of autophagy markers such as LC3II, p62, ATG5 and ATG12. In turn, CHOP promotes apoptosis by modulating the expression of Bax and Bcl-2 and activating caspase-12 and caspase-3 [134,135]. Similar changes in apoptotic markers and increased apoptosis were observed after exposure to PS-NPs in a human hepatocellular carcinoma cell line (HepG2) [130]. In vitro models showed that PERK silencing or pharmacological alleviation of ER stress reduced apoptosis, confirming an important role for this pathway in PS-MP-induced hepatotoxicity [135].

For NPs, it has also been shown that particle surface charge may be an important determinant of toxicity. As PS particles degrades, environmental factors such as light, pH and microbial activity can modify the particle surface through oxidation and adsorption, resulting in the formation of charged groups [138]. One study showed that charged PS-NPs induced ER stress in hepatocytes, accompanied by excessive ROS generation and reduced glutathione synthesis due to inhibition of SLC7A11-dependent cystine uptake. As a consequence, increased ferroptosis was observed. Ferroptosis is an iron-dependent form of regulated cell death associated with inactivation of glutathione peroxidase 4 (GPX4) [138,184]. Activation of ferroptosis, triggered by disturbances in iron, amino acid and lipid metabolism, has also been observed after exposure to PS-MPs [141]. Studies on the pathogenesis of liver disease, conducted independently of exposure to MNPs, have shown that ferroptosis, characterized by excessive lipid peroxidation and redox imbalance, may contribute to the development of alcoholic liver disease, non-alcoholic steatohepatitis and drug-induced liver injury. At the same time, its induction may also have a potentially beneficial role in liver fibrosis and hepatocellular carcinoma [184]. Inert PS-NPs can be phagocytosed by endothelial cells and localize in lysosomes, where they form phagolysosomes. Due to their limited susceptibility to degradation, destabilization of the lysosomal membrane and release of lysosomal enzymes, including β-galactosidase, may occur, leading to disruption of cellular homeostasis and promotion of accelerated cell aging [138].

In summary, MNPs can induce ER stress in hepatocytes, leading to oxidative stress, mitochondrial dysfunction and activation of autophagy as well as various forms of regulated cell death. The extent of these effects may also depend on the physicochemical properties of the particles, including their surface charge.

### 4.4. Genotoxicity and Nuclear and Mitochondrial DNA Damage Induced by MNPs in the Liver

DNA damage promotes loss of genome stability, cellular dysfunction and progression of chronic liver disease [185]. Increasing experimental evidence suggests that MNPs can induce damage to genetic material in hepatocytes and one of the main proposed mechanisms is genotoxicity secondary to increased oxidative stress [159,160,161,162,163].

In a HepG2/THP-1 co-culture model, reflecting immune-dependent liver injury, exposure to PMMA-MP led to increased levels of ROS and 8-OHdG, indicating oxidative DNA damage [159]. Another study on HepG2 cells showed that exposure to PET-MP and PE-MP resulted in increased ROS levels, decreased mitochondrial membrane potential and reduced mtDNA integrity. These results support the concept of oxidative stress-induced DNA damage and highlight the role of mitochondrial dysfunction in the mechanisms of genotoxicity [160]. Additionally, in HepG2 cells, NPs derived from actual PET products induced DNA strand breaks as detected by the comet assay, confirming their genotoxic potential in models similar to environmental exposure [161]. In HuH-7 cells both PET-NP and PLA-NP were shown to exacerbate oxidative stress, but only PLA-NP caused a significant increase in the number of DNA strand breaks in the comet assay [162].

The significance of these observations is supported by in vivo studies. Shen et al. showed that PS-MPs induced both nuclear and mitochondrial DNA damage, leading to translocation of dsDNA fragments into the cytoplasm and activation of the cGAS/STING pathway. This response is followed by activation of NF-κB, increased expression of pro-inflammatory cytokines and exacerbation of liver fibrosis [163]. Another study, conducted in a freshwater fish model (*Danio rerio* and *Perca fluviatilis*), showed that after exposure to PS-MPs, DNA damage was the most sensitive biomarker of toxicity in the liver of both species and these changes were accompanied by features of oxidative stress, induction of apoptosis and autophagy [175]. In addition, a study by Jeyavani et al. indicates that the degree of DNA damage may be dose and exposure time-dependent [128].

Taken together, the available data indicate that DNA damage is one of the main mechanisms of MNP-induced hepatotoxicity.

### 4.5. Effects of MNPs on Hepatic Inflammation

Experimental studies suggest that MNP exposure may be associated with hepatic inflammatory responses through several interconnected molecular mechanisms, including disruption of redox homeostasis, activation of the NLRP3 inflammasome, TLR-dependent signaling and changes in cytokine and chemokine profiles. Together, these processes may contribute to the persistence of inflammation and progression of liver injury [88,123,129,134,139,140,141,142,143,144,145,146,147,148].

One of the mechanisms by which MNPs may promote liver inflammation is activation of the NLRP3 inflammasome. In a mouse model of exposure to PS-NPs, suppression of the antioxidant NRF2 pathway was accompanied by increased expression of NLRP3, caspase-1 and IL-1β, suggesting a close association between redox imbalance and activation of the inflammatory response [88]. Endoplasmic reticulum stress-dependent activation of the NLRP3 inflammasome has also been described in carp exposed to PS-MPs [134]. In a number of studies, exposure to MNPs was associated with NLRP3 activation accompanied by increased hepatocyte pyroptosis [139,140,141]. Pyroptosis is a highly pro-inflammatory form of regulated cell death associated with inflammasome activation, caspase-1 maturation, and processing of IL-1β and IL-18. It may also promote activation of hepatic stellate cells (HSCs) and the development of fibrosis [186,187].

Activation of TLRs also plays an important role [142,143,144,145]. One possible mechanism for their stimulation may be increased translocation of lipopolysaccharide (LPS) from the gut into the circulation as a result of MNP-induced dysbiosis, indicating the involvement of the gut–liver axis [139,140,141,142,143,144]. LPS mainly activates TLR2 and TLR4 in the liver, leading to activation of the NF-κB pathway and secondary activation of the NLRP3 inflammasome and GSDMD, which further enhances inflammation [139,142,143,144].

Activation of NF-κB results in increased production of pro-inflammatory cytokines such as TNF-α, IL-1β and IL-6, which may be accompanied by increased infiltration of neutrophils and macrophages into liver tissue [123,143,145]. In some studies, this effect was attenuated after the use of probiotics (e.g., *Lactobacillus rhamnosus GG*, *Lactiplantibacillus plantarum ZP-6*), further highlighting the importance of the gut–liver axis [171,188]. Increased expression of TNF-α, IL-1β and IL-6 after MPs exposure has also been confirmed in other studies, although without a detailed explanation of the mechanism [129,146]. These cytokines are known mediators of hepatocyte injury, inflammatory amplification, hepatic stellate cell activation and are implicated in the progression from steatosis to steatohepatitis [189].

Exposure to MNPs also affects the chemokine profile and inflammatory cell influx. The increase in IL-17A levels observed after exposure to PS-MPs leads to activation of NF-κB and, consequently, increased expression of cytokines and chemokines [150]. Increased expression of *Ccl2* (MCP-1) and *Ccl5* has been shown in mice exposed to MPs [147,148]. These chemokines are involved in the recruitment of inflammatory cells and, at the same time, can activate HSCs, creating a link between chronic inflammation and the fibrotic process [190]. In the case of NPs, increases in chemokines such as CXCL1, CXCL2, CCL2 and CXCL12 have been observed primarily in the presence of additional factors such as choline-deficient L-amino acid defined high-fat diet (CDAHFD) or co-exposure to glyphosate [191,192].

Increased secretion of pro-inflammatory cytokines may also indicate a shift in macrophage polarization towards the M1 phenotype, as described in mice exposed to MPs [150,193]. Under conditions of liver injury, the balance between M1 and M2 macrophages is important for the course of the inflammatory response. Predominance of M1 macrophages promotes ongoing inflammation and exacerbates tissue damage, whereas M2 macrophages contribute to resolution of inflammation and support tissue repair. Simultaneously, the downregulation of anti-inflammatory mediators linked to the M2 phenotype, including IL-10 and TGF-β, has been reported [150,176]. Similar trends have been observed after exposure NPs, but the available data are mainly from extrahepatic models, so their relevance to liver processes still requires further investigation [192,194].

Finally, MNPs can induce extracellular trap formation. MPs have been shown to stimulate macrophage extracellular trap (MET) formation through phagocytosis and lysosomal damage, whereas NPs may induce neutrophil extracellular trap (NET) formation by increasing chemokines such as CCL2 and CXCL12 [164,172]. These extracellular DNA–protein structures can sustain sterile inflammation and contribute to tissue damage, partly through ROS/NLRP3- and TGF-β/Smad-related pathways [164,172,195,196,197].

In summary, experimental studies suggest that MNP exposure may be associated with hepatic inflammation, involving redox imbalance, inflammasome activation, TLR-dependent signaling, and altered cytokine and chemokine profiles. These changes may contribute to sustained inflammatory responses and liver injury progression.

### 4.6. Effects MNPs on Lipid Metabolism

#### 4.6.1. Enhanced De Novo Lipogenesis and Hepatic Lipid Accumulation

Experimental studies suggest that MNP exposure may affect transcription factors involved in fatty acid synthesis and promote lipid accumulation in hepatocytes, a process relevant to hepatic steatosis [127,129,137,149,150,151,152,153]. The available results indicate that PS-MPs increase the levels of the transcription factor sterol regulatory element-binding protein 1 (SREBP1) as well as the activity of acetyl-CoA carboxylase 1 (ACC1) and fatty acid synthase (FASN) in the liver. These enzymes play an important role in the regulation of de novo lipogenesis and their excessive activation is associated with lipid accumulation and the development of hepatic steatosis [150,198]. Consistent findings were obtained in another study, in which increased SREBP1 expression and elevated ACC and FASN activity were observed alongside greater severity of hepatic steatosis in mice [127].

Furthermore, Del Piano et al. observed elevated levels of peroxisome proliferator-activated receptor gamma (PPARγ), in addition to increased expression of SREBP1 and FASN. PPARγ is a nuclear receptor involved in the regulation of hepatic lipid metabolism, and its overexpression has been associated with hepatic steatosis [129]. Similar disturbances in lipid metabolism have also been described following exposure to NPs. In mouse studies, exposure to PS-NPs was shown to increase the expression of lipogenesis-related genes and enhance lipid accumulation in hepatocytes. This mechanism may be related to activation of endoplasmic reticulum stress and the PERK–ATF4 pathway, which in turn increases the expression of genes regulating lipid synthesis, including SREBP1 and PPARγ [137,149]. Exposure to UV-aged PS-NPs was also associated with comparable changes [151]. On the other hand, another study of PS-NP exposure showed that increased hepatic lipid accumulation was associated with inhibition of the PPARγ pathway together with increased SREBP1 activity, suggesting a more prominent role of SREBP1 in PS-NP-induced lipogenesis [152]. Taken together, these data suggest that MNPs may affect similar pathways regulating de novo lipogenesis and lipid deposition in hepatocytes.

In addition, exposure to PS-NPs may lead to disturbances in hepatic lipid metabolism by upregulating the expression of genes related to fatty acid esterification (*Dgat1*, *Dgat2*) and lipid transport (*ApoB*, *Cd36*, *ApoE*), as well as by increasing hepatic triglyceride and phospholipid concentrations [153]. PS-NPs were also associated with increased hepatic lipid deposition and alterations in CYP-related lipid pathways, including linoleic acid, glycerophospholipid, and arachidonic acid metabolism [199]. Independent studies have shown that arachidonic acid metabolites play an important role in the progression and severity of MAFLD and liver fibrosis [200].

In conclusion, MNPs may enhance lipogenesis and lipid deposition in the liver through activation of SREBP1 and related metabolic pathways. As a result, they may promote the development of steatosis and the progression of fatty liver disease.

#### 4.6.2. Effects of Micro- and Nanoplastics on Lipid Catabolism

Lipid catabolism in hepatocytes involves several complementary processes, including hydrolysis of triglycerides accumulated in lipid droplets, autophagic lipid degradation (lipophagy) and mitochondrial and peroxisomal β-oxidation of fatty acids [201,202]. Experimental studies indicate that MNPs may interfere with these metabolic processes at different stages [150,154,155,156,157,158].

For example, exposure to MPs may reduce the expression of proteins involved in lipid metabolism, such as adipose triglyceride lipase (ATGL), acyl-CoA oxidase 1 (ACOX1) and carnitine palmitoyltransferase 1 alpha (CPT1α), thereby promoting lipid accumulation in the liver [150]. These proteins regulate triglyceride breakdown and mitochondrial or peroxisomal fatty acid oxidation [201,202,203]. Similar phenomena may also occur after exposure to NPs. In a model of dietary exposure to PS-NPs, excessive lipid deposition in the liver was observed in the large yellow croaker, accompanied by inhibition of the AMPK- peroxisome proliferator-activated receptor alpha (PPARα) pathway and decreased expression of lipolysis-related genes [154]. Lu et al. showed that lysosomal function and autophagic flux were impaired after exposure of mice to PS-NPs. These mechanisms are involved in the cellular degradation of lipid components, and their impairment led to increased lipid accumulation in the liver. The same study also indicated an important role of lysosomal exocytosis in the removal of intracellular PS-NPs [155].

Different results were presented by Zhang et al. who observed an increase in lipid catabolism after exposure to MPs. This was accompanied by an increase in the expression of PPARα, retinoid X receptor (RXR), CPT1α and ACOX1, which may indicate the activation of fatty acid oxidation processes in the liver of Japanese quail [156]. Furthermore, increased expression of long-chain acyl-CoA synthetase 1 (ACSL1) may have promoted the conversion of long-chain fatty acids to their acyl-CoA derivatives, which can then be oxidized in the mitochondria [156]. The increased lipid catabolism observed in the study by Zhang et al. may be compensatory to the parallel increase in triglyceride synthesis induced by exposure to MPs. At the same time, this process may be associated with increased ROS production and increased MDA levels [156,204]. Similarly, an increase in the expression of genes encoding lipid catabolism-related proteins such as ACOX1, CPT1α, APO3, angiopoietin-like 4 (ANGPTL4) and phosphoenolpyruvate carboxykinase (PCK) has been reported after exposure to NPs [155,157]. In some of these studies, there was a concomitant increase in the expression of lipogenesis-related genes such as *Srebf1*. This supports the hypothesis of a compensatory increase in lipid catabolism. However, this effect was observed only for particles with a diameter of 100 nm [155].

In contrast, the results of Ge et al. highlight the importance of the route of exposure in PS-NP-induced hepatotoxicity in mice. After oral administration of PS-NPs, a decrease in diacylglycerols (DG) and phosphatidic acids (PA) was observed, associated with an increase in lipid hydrolysis, whereas polyunsaturated fatty acids (PUFAs) and lipid peroxidation markers increased after inhalation exposure. Despite these differences, an increase in β-oxidation-related gene expression was observed in both groups [158]. There are also studies in which no significant changes in the expression of genes encoding proteins related to lipid catabolism, such as PPARα, hepatic lipase (HL), phospholipase A2 (PLA2) or fatty acid binding protein 1 (FABP1), which is involved in intracellular lipid transport in hepatocytes, were found after exposure to microplastics [129].

In summary, the effects of MNPs on lipid catabolism appear to be ambiguous and may depend on the research model, as well as the time, dose, and route of exposure (Table 3 and Table 4).

### 4.7. Liver Fibrosis

Liver fibrosis results from chronic liver injury and is mainly driven by hepatic stellate cell activation, extracellular matrix deposition, inflammation, oxidative stress, hepatocyte injury, and profibrotic cytokines such as TGF-β [186]. Available evidence suggests that MNPs may enhance several of these processes involved in fibrogenesis [147,164,165,166,167,168].

In one study, this effect of exposure to PS-MNPs was linked primarily to increased mRNA expression of pro-inflammatory cytokines such as *Il-6*, *Il-1β* and *Tnf-α*, as well as increased lipid deposition in the livers of mice [165]. At the same time, the expression of genes such as *Acot3*, *Abcc3*, *Nr1* and *Nr3* was also increased. These genes are involved in fatty acid metabolism, transmembrane transport and cellular responses to xenobiotics. Taken together, these findings suggest that disruption of metabolic and inflammatory pathways may link MNP exposure with fibrogenic changes in experimental models. In contrast, no significant contribution of oxidative stress was demonstrated in this model [165]. Another study, in which hepatocyte swelling, increased inflammation and hepatic collagen deposition were observed in mice after exposure to PET-MPs, also found elevated levels of oxidative stress markers and increased expression of proteins related to the p38 MAPK/p65 NF-κB signaling pathway [166]. Importantly, both administration of N-acetylcysteine, which reduces oxidative stress, and inhibition of p38 MAPK activation attenuated liver fibrosis, indicating an important role of these mechanisms in its development [166]. Similar findings were reported by Park et al., who found that mice exposed to PET-MPs developed hepatomegaly, hepatic steatosis and early signs of fibrosis. This was supported by increased expression of fibrosis-related genes, including *Col1a1*, *Col3a1*, *Fn1* and *Acta2* (α-SMA) [167].

Djouina et al. showed that MP exposure increased *Pdgfa* expression, a marker associated with HSC activation, which was consistent with increased hepatic collagen deposition [147,205]. In addition, MPs exacerbated fibrosis following exposure to carbon tetrachloride (CCl_4_), a potent environmental toxin emitted by the chemical industry that exerts hepatotoxic effects through free radical-dependent inflammatory processes [147,206]. Another study showed that NPs can activate the PDGFRα-PI3K signaling pathway, which is involved in cell differentiation toward a myofibroblast phenotype. Simultaneous exposure to NPs and dibutyl phthalate (DBP), a common environmental plasticizer, further led to a synergistic enhancement of hepatotoxicity and liver fibrosis progression, indicating an important role for interactions between different environmental contaminants [168].

The Wnt/β-catenin signaling pathway has also been shown to play an important role in the progression of liver fibrosis induced by exposure to MPs in diabetic mice [177]. This pathway can be activated by ROS and plays an important role in regulating fibroblast activation, proliferation and differentiation into myofibroblasts, which are responsible for the production of ECM components [177]. Another pathway that may be involved in the development of MAFLD and liver fibrosis is the Hedgehog signaling pathway, the activation of which was found to be increased in hepatocytes from mice exposed to NPs and fed a CDAHFD. The observed activation of Sonic Hedgehog signaling in HSCs promoted the development of liver fibrosis [192].

### 4.8. MNPs and the Multiple-Hit Hypothesis in MAFLD

Currently, one concept explaining the development of MAFLD is the multiple-hit hypothesis, which emphasizes the importance of genetic and epigenetic factors, as well as factors that may be influenced by MNPs. These include endoplasmic reticulum stress, mitochondrial dysfunction, increased hepatic inflammation, dysregulated lipid metabolism and intestinal dysbiosis, as discussed previously [6,88,123,124,125,126,127,128,129,134,137,139,141,142,143,144,145,146,147,148,151,153,154,155,156,157,158,198,207].

In addition, insulin resistance is considered one of the main factors initiating and sustaining the development of MAFLD and it can be induced by both MNPs and NPs [149,208,209,210,211]. In MP exposure models, disruption of the gut–liver axis may involve increased permeability of the intestinal barrier, translocation of lipopolysaccharide (LPS) into the circulation and an increased inflammatory response involving cytokines such as TNF-α and IL-1β. This process may result in inhibition of insulin receptor substrate 1 (IRS-1) and decreased expression of phosphoinositide 3-kinase (PI3K), proteins involved in insulin signal transduction. These changes may promote the development of insulin resistance [209,210]. NPs can also induce insulin resistance, including through the activation of the pro-inflammatory NF-κB and MAPK signaling pathways and by increasing oxidative stress, but these studies did not analyze the role of the gut–liver axis [149,211].

PS-NPs have also been shown to enhance liver fibrosis in high-fat diet-induced NAFLD models, accompanied by increased oxidative stress, enhanced inflammatory response and hepatic steatosis [212]. Furthermore, experimental data suggest that NPs may exacerbate mechanisms involved in the transition from simple steatosis (NAFL) to NASH, including oxidative stress, inflammation, lipid uptake and fibrogenic processes. In this process, an important role has been attributed to impaired endoplasmic reticulum-mitochondria contacts, known as mitochondria-associated membranes (MAMs), and disruption of the Nrf2/miR26a axis [173].

Taken together, the available experimental data suggest that MNPs may aggravate the development of MAFLD by affecting oxidative stress, inflammation, metabolic disorders and fibrosis, as well as promoting insulin resistance.

### 4.9. Cholestasis and Dysregulation of Bile Acid Metabolism by MNPs

Bile acid homeostasis depends on the balance between its synthesis in hepatocytes, secretion into bile, intestinal metabolism and enterohepatic circulation [213]. Disruption of any of these steps can lead to cholestasis, intrahepatic bile acid retention and secondary damage to hepatocytes and intestinal dysbiosis may play an important role in exacerbating these disorders [214].

A growing body of experimental data suggests that MNPs can disrupt this system at multiple levels. For example, one study showed that exposure to PS-MPs led to cholestasis and an altered ratio of primary to secondary bile acids in the feces. This phenomenon was accompanied by damage to the intestinal barrier and changes in the composition of the microbiota, suggesting disruption of bile acid metabolism through the gut–liver axis [178]. Other studies, however, a direct effect of MPs on bile acid metabolism is indicated by a study by Yang et al. in which exposure to polylactide (PLA) led to increased expression of fibroblast growth factor receptor 4 (FGFR4), activation of the c-Jun N-terminal kinase (JNK)/ERK pathway and subsequent inhibition of CYP7A1, a key enzyme in bile acid synthesis. These changes were linked to a decrease in bile acid synthesis, a disturbed bile acid profile and increased inflammation and liver damage. This indicates an important role for these pathways in PLA-induced hepatotoxicity [215]. In contrast, other studies suggest that PS-NPs may transiently increase bile acid synthesis by interfering with the lysosomal degradation of CYP7A1. Increased bile acid levels have been associated with adverse changes in the gut microbiota and increased susceptibility to colitis in animal models [78]. In addition, it has been shown that PS-MPs can accumulate in bile duct structures and that their elimination from hepatocytes depends on the activity of the BSEP and MRP2 bile transporters. In studies using human hepatobiliary organoids (HBOs), inhibition of these transporters exacerbated hepatotoxicity, whereas increased biliary secretion promoted the removal of MPs particles. These results suggest that biliary secretion may be an important mechanism of MP elimination from the liver and that its impairment may exacerbate damage to this organ [179].

### 4.10. MNPs, Heavy Metals and Liver Injury

An increasing number of studies show that MNPs can interact with heavy metals and thus exacerbate their toxic effects on the liver. This is important because in the environment, contaminants do not usually occur separately. MPs can bind metals on their surface and act as carriers, increasing their bioavailability and promoting bioaccumulation in organisms [1,11].

The best-described example of such an interaction is cadmium in combination with MNPs. In an in vitro study, concurrent exposure to cadmium (Cd) and MPs was shown to enhance inflammation and liver fibrosis via activation of the ATP–P2X7 pathway [216]. The P2X7 receptor can activate the NLRP3 inflammasome, which leads to increased release of pro-inflammatory cytokines and enhanced fibrogenesis [186,187]. Similar results have been observed with polyvinyl chloride (PVC) MPs, which, in the presence of cadmium, increase oxidative stress, promote lipid accumulation in the liver and worsen fibrosis and apoptosis in hepatocytes [217].

In a separate study, concurrent exposure to PS-MPs and Cd was shown to reduce the activity of CPT1, an enzyme crucial for the β-oxidation of fatty acids, resulting in increased lipid accumulation [218]. Dysbiosis of the intestinal microbiota and the accompanying increase in intestinal barrier permeability may also play an important role in the synergistic toxic effects of PS-MPs and Cd [219]. This mechanism may explain the greater accumulation of NPs in the liver and the more severe damage to this organ observed with simultaneous exposure to cadmium and NPs [136,220,221,222]. One study further suggested that, although 1 μm PS particles were more hepatotoxic alone, 100 nm NPs more strongly enhanced Cd-induced liver damage, possibly due to easier cellular internalization and more direct interactions with Cd ions [136]. Similar findings have been observed with other heavy metals. In a study of co-exposure to arsenic (As) and NPs, it was shown that PS-NPs could induce excessive activation of autophagy, apoptosis, and pyroptosis in the liver by modulating the PI3K–mTOR and NLRP3/Caspase-1 pathways and altering the expression of apoptotic markers; significant toxic effects only appeared with simultaneous exposure to both agents [223]. In another experimental model, PS-NPs were found to promote arsenic accumulation in *Danio rerio* tissues, with the highest efficiency of this process observed in the liver, and the presence of NPs further enhanced As-induced oxidative stress [224].

In the case of mercury (Hg), it has been shown that the presence of MPs can increase mercury bioaccumulation in the liver and exacerbate oxidative stress. In juveniles of *Dicentrarchus labrax*, simultaneous exposure to MPs and Hg led to additive or synergistic increases in its concentration in the liver and marked redox imbalances [225]. In another study, PS-MPs increased methylmercury (MeHg) bioaccumulation in tissues of zebrafish embryos, including the liver and increased oxidative stress markers [226]. The induction of abnormalities in the composition of the gut microbiota by NPs and Hg may also the function of the gut–liver axis and the development of hepatic damage [227].

Increased hepatotoxicity was also observed during co-exposure to lead (Pb) and MPs. In one study, PVC-MPs or PE-MPs in combination with Pb caused more severe damage to hepatocytes than exposure to either agent alone and also led to worsened insulin resistance; these changes were linked to activation of the Nrf2/NF-κB signaling pathway [228].

Copper (Cu), although an essential cofactor of numerous enzymes, may exhibit hepatotoxic effects at elevated concentrations, including by exacerbating oxidative stress. Accumulation of Cu^2+^ ions is particularly high in the liver [229]. It has also been shown that PVC-MPs can enhance hepatic copper accumulation in *Sebastes schlegelii*, which has been linked to the increased hepatotoxicity observed during simultaneous exposure to Cu^2+^ and PVC-MPs [230]. In addition, exposure to Cu^2+^ in combination with PS-MPs may lead to an increase in the abundance of potentially pathogenic bacteria in the intestine, thereby favoring increased intestinal barrier permeability. This phenomenon may facilitate copper absorption and enhance copper accumulation in the liver, as reflected in the histopathological changes observed during this co-exposure [229]. In turn, another study showed that simultaneous exposure to PLA-NPs and copper led to ferroptosis and liver fibrosis. The authors linked this effect to disturbances in the function of the gut–liver axis [231].

In conclusion, the experimental findings indicate that MNPs may intensify the hepatotoxic effects of heavy metals through various mechanisms. MNPs may increase heavy metal bioaccumulation, disrupt the intestinal barrier, increase oxidative stress and activate pathways linked to inflammation and fibrosis. This demonstrates the importance of studying mixtures of environmental contaminants, as in real-world conditions organisms are usually exposed to mixtures of contaminants rather than to single substances.

**Figure 2:**
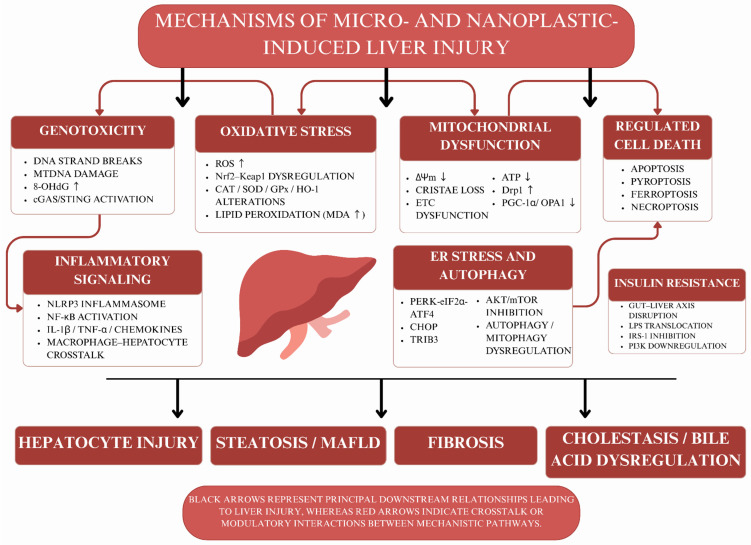
Schematic summary of mechanisms of micro- and nanoplastic-induced liver injury (figures were prepared using Canva Pro). Arrows indicate the direction of change (↑ increase; ↓ decrease). Solid, black arrows indicate the principal downstream relationships between major pathogenic mechanisms and hepatic outcomes. Curved/red arrows indicate crosstalk or modulatory interactions between mechanistic pathways. The scheme summarizes the main processes discussed in this review, including oxidative stress, mitochondrial dysfunction, inflammatory signaling, endoplasmic reticulum stress/autophagy dysregulation, genotoxicity, regulated cell death and insulin resistance, which may contribute to hepatocyte injury, steatosis/MAFLD, fibrosis and cholestatic/bile acid disturbances. ROS—reactive oxygen species; Nrf2—nuclear factor erythroid 2-related factor 2; Keap1—Kelch-like ECH-associated protein 1; CAT—catalase; SOD—superoxide dismutase; GPx—glutathione peroxidase; HO-1—heme oxygenase-1; MDA—malondialdehyde; mtDNA—mitochondrial DNA; cGAS—cyclic GMP-AMP synthase; STING—stimulator of interferon genes; ΔΨm—mitochondrial membrane potential; ETC—electron transport chain; ATP—adenosine triphosphate; Drp1—dynamin-related protein 1; PGC-1α—peroxisome proliferator-activated receptor gamma coactivator 1-alpha; OPA1—optic atrophy 1; PERK—protein kinase R-like endoplasmic reticulum kinase; eIF2α—eukaryotic initiation factor 2 alpha; ATF4—activating transcription factor 4; CHOP—C/EBP homologous protein; TRIB3—tribbles pseudokinase 3; AKT—protein kinase B; mTOR—mechanistic target of rapamycin; NLRP3—NOD-like receptor family pyrin domain containing 3; NF-κB—nuclear factor kappa B; IL-1β—interleukin 1 beta; TNF-α—tumor necrosis factor alpha; MAFLD—metabolic dysfunction-associated fatty liver disease; 8-OHdG—8-hydroxy-2′-deoxyguanosine; LPS—lipopolysaccharide; IRS-1—insulin receptor substrate 1; PI3K—phosphoinositide 3-kinase.

Taken together, these findings indicate that MNP-induced liver injury results from a network of interconnected mechanisms, including oxidative stress, mitochondrial dysfunction, endoplasmic reticulum stress, genotoxicity, inflammatory signaling and regulated cell death. These mechanisms collectively promote hepatocyte injury, steatosis, cholestatic disturbances, and fibrosis (Figure 2).

### 4.11. Differences in the Effects of MNP Polymer Types

The effects of PS-MNPs on the liver are the best documented. PS-MNPs have most frequently been associated with a broad spectrum of changes, including oxidative stress, mitochondrial dysfunction, activation of inflammatory pathways, increased lipogenesis and fibrosis, and impaired bile acid metabolism [88,124,125,127,130,131,136,140,155,174,178,179].

Data on the other polymers are less abundant and primarily concern MPs [142,147,160,166,167,232]. However, the available evidence suggests polymer-specific differences in the induced hepatic effects. For PE-MPs, activation of the TLR2/NF-κB/NLRP3 inflammatory pathway in the liver was mainly described [142]. In contrast, for PET-MPs, mitochondrial damage reduced mtDNA integrity and activation of inflammation involving the p38 MAPK/p65 NF-κB axis were more frequently indicated [160,166,167]. The results obtained in the HepG2 cell model further suggest that both particle types can increase ROS production, disrupt mitochondrial function and activate autophagy, but PET-MPs may have stronger effects (Table 4) [160].

For PP-MPs, the main reported effects involve disruption of cellular bioenergetics, including a decrease in the activity of respiratory chain complexes, a decrease in ATP production and an increase in reductive stress, especially after exposure to aged particles [6,128]. Similarly, in vitro, PMMA-MPs have been associated with increased oxidative stress and inflammatory responses in hepatic cells, accompanied by impaired lipid metabolism [159].

Few in vivo studies directly comparing the effects of PE-, PP-, PS- and PET-MPs on the liver are available. One study in mice showed that all four polymers could impair liver function, increase oxidative stress and the inflammatory response, even in the absence of clear histopathological changes. The strongest effects were observed after exposure to PS- and PET-MPs, which the authors linked to the presence of benzene rings in the structure of these polymers [233]. However, there is still a lack of comparative studies conducted under standardized exposure conditions to clearly delineate polymer-specific differences between MP types.

For NPs, data on polymers other than PS-NPs remain limited. Studies on PET-NPs indicate that these particles are associated with mitochondrial damage and genotoxicity, including a decrease in mtDNA integrity and an increase in DNA strand breaks in liver cell models, which remains consistent with effects previously described for PET-MPs [160,161,162,166]. In addition, one study on HepG2 cells showed greater cytotoxicity of PET-NPs and PVC-NPs than of PS-NPs, associated with increased levels of intracellular ROS, enhanced apoptosis and inhibition of DNA repair gene expression in the p53 pathway. Differences in the density and chemical structure of the tested polymers were proposed as possible explanations for these differences [234]. A similar relationship was described in the HUH-7 cell model, in which unmodified PS-NPs and their carboxylated derivatives did not induce significant changes, whereas exposure to PET-NPLs and PLA-NPLs was associated with enhanced ROS production, genotoxicity and increased cytokine secretion [162]. This indicates that polymer properties may also significantly modulate the hepatic cell response to NPs, although these observations still need to be confirmed in in vivo studies.

### 4.12. Translational Limitations of Studies on MNP-Induced Hepatotoxicity

Many in vitro and in vivo studies have used MNP concentrations or doses spanning several orders of magnitude, for example, from 50 µg/mL to 1 mg/mL in vitro and from 0.05 to 100 mg/kg/day or higher in vivo, which makes direct comparison with real-world environmental exposure difficult [88,140,159]. Estimates of human exposure remain highly variable. Population-level data suggest an intake of approximately 39,000–52,000 MP particles/year through diet and 74,000–121,000 particles/year when inhalation is included [235]. Mass-based estimates, in turn, suggest a possible intake of 0.1–5 g/week, corresponding to approximately 0.2–10.2 mg/kg/day for a 70 kg individual [236].

However, physiologically based pharmacokinetic models indicate that the predicted tissue burden of retained particles may be substantially lower than external exposure estimates. The estimated median intake in adults is 883 particles/day, corresponding to 583 ng/day, whereas the modeled lifetime tissue accumulation up to 70 years of age is approximately 50,100 particles, corresponding to 40.7 ng [237]. Quantitative data on human exposure to NPs remain more limited. Available analytical studies indicate that bottled water may contain MNPs at approximately 10 ng/L, with NPs accounting for approximately 90% of detected particles [238]. In mass-based terms, this estimated concentration is several orders of magnitude lower than the concentrations used in many experimental studies [88,125,130,131,132].

These data support the plausibility of chronic human exposure to MNPs, but do not allow direct extrapolation of findings from high-dose in vitro and in vivo models to typical environmental exposure scenarios [1,3,4,5]. This limitation is further highlighted by the conclusions presented in Section 4.11, as the best-documented hepatotoxic effects relate mainly to PS-MNPs, whereas environmental MNPs represent heterogeneous mixtures differing in polymer composition, size distribution, shape, aging status, surface properties, and the presence of chemical additives and adsorbed contaminants [169]. Therefore, the widespread use of monodisperse polystyrene microspheres raises concerns regarding their suitability as models of environmentally relevant MNPs [239].

Current evidence supports the biological plausibility of adverse molecular responses in the liver, including oxidative stress, inflammatory activation, mitochondrial dysfunction, and disrupted lipid metabolism. However, the available data remain insufficient for precise quantitative risk assessment in humans under typical environmental exposure conditions [88,124,125,127,130,131,136,140,155,174,178,179]. Future studies should focus on chronic low-dose exposure models and incorporate aged, irregularly shaped particles and mixed-polymer MNP preparations.

## 5. Discussion

By bringing together exposure routes, systemic distribution, analytical detection, and hepatotoxic mechanisms, this review highlights an important interpretative issue: the biological plausibility of MNP-related liver injury is supported by several converging lines of evidence, but these lines differ substantially in their strength and translational relevance. Human studies mainly confirm exposure and tissue accumulation, whereas mechanistic evidence for liver injury still comes predominantly from experimental models. Therefore, the available literature should be interpreted as a continuum of evidence rather than as direct proof of causality in humans.

However, several limitations of the current evidence should be emphasized. Human studies mainly show that MNPs can be detected in blood and tissues, including the liver, but this should not be interpreted as direct proof of causality. Causal links with liver injury are still inferred mostly from experimental models. In addition, many available studies use high doses and simplified exposure systems, which may not reflect chronic, low-level human exposure.

A further major limitation is the predominance of spherical, monodisperse polystyrene particles in experimental studies. Although useful for controlled mechanistic research, such models only partially reflect real-world exposure, which includes mixed polymer types, irregular and aged particles, plastic additives, biofilms or ecocoronas, and adsorbed pollutants such as heavy metals and persistent organic compounds. Future studies should therefore use more environmentally relevant mixtures of particles, realistic exposure levels and standardized detection methods, as well as clearer reporting of particle size, shape, polymer type, dose, and biological outcomes.

## 6. Conclusions

MNPs should no longer be viewed only as passive environmental pollutants. The available evidence suggests that they are biologically active particles that may be relevant to liver pathology. Their importance in hepatology is supported not only by their detection in human biological samples and organs, but also by experimental findings showing that they can affect several key processes involved in liver injury, including oxidative balance, mitochondrial function, endoplasmic reticulum stress, inflammatory signaling, lipid metabolism and fibrogenic responses. At the same time, the current evidence is stronger on the mechanistic side than on the clinical side. Human studies increasingly support systemic exposure and tissue deposition, but they are still limited by major methodological differences, insufficient standardization and the difficulty of reliably detecting the smallest particle fractions. For this reason, MNPs can currently be regarded as plausible contributors to hepatic injury, but not yet as independent and clearly established drivers of human liver disease.

An important conclusion from this review is that progress in this field will require closer integration of exposure science, analytical chemistry, experimental hepatology and human biomonitoring. A better understanding of the full-body kinetics of MNPs is also needed. Future studies should clarify the relative importance of different exposure routes, the efficiency of barrier crossing, the pathways of systemic transport and the factors that determine tissue-specific retention and clearance. Standardized detection methods, clearer separation of micro- and nanoscale fractions and studies linking tissue burden with clinically relevant liver outcomes will be necessary to determine whether MNPs act mainly as direct toxicants, cofactors that worsen existing liver injury, or both. Although direct clinical evidence remains limited, the current findings suggest that reducing avoidable exposure to micro- and nanoplastics may be particularly important in individuals with pre-existing liver disease or increased susceptibility to hepatic injury.

## Figures and Tables

**Table 1:** Relative strength of evidence for major MNP exposure and entry routes.

Exposure Route	Relative Strength of Evidence	Main Supporting Evidence	Main Limitations
Gastrointestinal	Strongest	Dietary exposure data, intestinal barrier models, epithelial translocation mechanisms, and possible access to portal or lymphatic pathways [20,22,25,26,27,28,29,30,31,32,33,34,35,36,37,38,39,40,41,42,43,44,45,46,47,48,49]	Direct human evidence for intestinal translocation remains limited
Respiratory	Moderate	Airway deposition, epithelial uptake, animal/in vitro translocation studies, and extrapulmonary distribution after respiratory exposure [50,51,52,53,54,55,56,57,58,59]	Human evidence for efficient alveolar-to-blood passage remains limited
Dermal	Limited/weak	Local penetration shown mainly in ex vivo human skin and reconstructed 3D skin models, especially under impaired barrier conditions [23,24,60,61]	No convincing direct evidence of clinically relevant skin-to-blood passage in humans
Iatrogenic/direct bloodstream	Plausible but insufficiently quantified	Particle release or contamination reported in infusion fluids, PVC tubing, infusion bags, infusion sets, and needles [62,63,64,65,66,67]	Actual dose transferred to patients, tissue retention, and clinical relevance remain uncertain

**Table 2:** Summary of analytical methods used for MNP detection in biological samples.

Method	Sample Type	Detection Range	Strengths	Limitations
FTIR/µFTIR	whole blood [91], placenta [93], stool [94], urine [95], sputum [96], lung [97], colon [98], synovial tissue [99]	mostly MPs, usually ~10–20 µm [92]	rapid, reproducible, and relatively low-cost polymer identification [92]	susceptible to background and matrix interference; limited sensitivity for NPs [92]
Raman spectroscopy	liver [87], spleen [87], kidney [87], placenta [103], breast milk [104], semen [105], stool [106], urine [107], lung tissue [108]	lower µm range, approximately ≥1 µm in selected biological samples [103]	higher spatial resolution than FTIR, useful for polymer identification of smaller particles in complex biological tissues [102]	fluorescence, slow analysis [102]
Py-GC-MS	blood [110], testes [111], semen [111]	polymer mass, not particle-resolved [90]	good quantification of polymer mass [90]	no size, shape, number, localization [90]
Microscopy	biological samples, cells, and tissues [72,115]	mainly >1 µm by light microscopy; submicron-to-nanometric morphology by SEM, TEM, or AFM [72,115,116,117]	rapid visualization of morphology, size, localization, and cellular uptake; useful complementary screening tools [72,114,115]	limited quantification; possible false positives with fluorescence/Nile Red staining [114,115,116]
Advanced nanoplastic-focused techniques: AFM-IR, O-PTIR, DLS, NTA	suspensions, isolated particles, and selected tissue/biological samples after specialized preparation [84,85,118]	AFM-IR: ~20 nm resolution; O-PTIR: submicron range; DLS/NTA: nanoparticle-size suspensions, including sub-20 nm particles [84,85,118]	analysis of very small MNPs; AFM-IR/O-PTIR—high-resolution chemical or photothermal information; DLS/NTA—particle size distribution and particle number in suspension [84,85]	requiring complementary validation; DLS/NTA lack polymer identification and are limited in complex biological matrices [85]

**Table 3:** Selected studies on the effects of NPs on the liver.

Reference	Type of NP, Size [nm]	Experimental Model	Dose, Route of Exposure, Exposure Duration	Main Outcomes After NPs Exposure *
Wen et al., 2024 [88]	PS, 20	Kunming mice	0.05, 0.5 or 5 mg/kg/day, oral (water), 28 days	1. ↑ ROS production (in mice and in AML-12) 2. ↓ NRF2 antioxidant pathway activity (in mice: ↑ KEAP1 and ↓ NRF2; in AML-12: ↓ NRF2 and ↓ p-NRF2) 3. ↓ Downstream antioxidant enzyme expression (in mice: ↓ GSH and ↓ CAT; in AML-12: ↓ HO-1) 4. ↑ NLRP3, ↑ IL-1β, ↑ caspase-1 expression and ↑ NF-κB activation (in mice); ↑ NLRP3 and ↑ caspase-1 (in AML-12) 5. Hepatocellular inflammatory injury (in mice and in AML-12)
		Alpha mouse liver-12 (AML-12) hepatocytes	50, 100 or 150 μg/mL, 24 h	
Guo et al., 2024 [125]	PS, 20	HepG2 and L02 cells	0–50 μg/mL, 12 or 24 h	1. ↑ Mitochondrial damage; effect a more severe effect in HepG2 cells 2. ↑ Inflammatory signaling, with an earlier and stronger response in HepG2 cells 3. ↑ ROS and NRF2 pathway activation in HepG2 cells
Li et al., 2023 [130]	PS, 20	HepG2 cells	6.25, 12.5, 25 or 50 μg/mL, 24 h	1. ↓ Cell viability 2. ↑ Mitochondrial injury and ROS production 3. ↑ Apoptosis through mitochondrial signaling 4. ↑ Mitochondrial fission, with altered DRP1/OPA1/PGC-1α-related regulation
Fan et al., 2024 [131]	PS, 20 and 100	Wild-type BALB/c mice	60 mg/kg, intraperitoneal injected, 24 h	1. ↑ Mitochondrial accumulation of 20 nm PSNPs in macrophages (in RAW 264.7 and J774A.1) 2. ↑ Macrophage necroptosis induced selectively by 20 nm PSNPs, rather than larger particles (in RAW 264.7; cytotoxicity also confirmed in BMDMs, peritoneal macrophages, J774A.1 and THP-1) 3. ↓ Mitochondrial integrity (in RAW 264.7) 4. ↑ mtROS production (in RAW 264.7) 5. ↑ Macrophage–hepatocyte crosstalk leading to hepatocyte injury (in RAW 264.7–AML-12 co-culture; supported in vivo by macrophage depletion in mice) 6. ↑ Acute liver injury in mice
		RAW264.7 macrophages, BMDMs, peritoneal macrophages, J774A.1, THP-1, AML-12 co-culture	50 μg/mL, 4 h	
Lin et al., 2022 [132]	PS, 80	Hepatic L02 cells	0.006, 0.0125, 0.03125, 0.0625, 0.125 and 0.25 mg/mL, 48 h	1. ↑ Mitochondrial damage (in L02 and BEAS-2B; effect more pronounced in L02) 2. ↑ mtROS production (in L02 and BEAS-2B; more marked in L02 than in BEAS-2B) 3. ↑ Mitochondrial-related metabolic disruption (in L02 and BEAS-2B) 4. Metabolic function was more vulnerable in hepatic L02 cells than in lung BEAS-2B cells (especially at low NPs concentrations)
		Human lung epithelial BEAS-2B cells	0.006, 0.0125, 0.03125, 0.0625, 0.125 and 0.25 mg/mL, 48 h	
Yu et al., 2024 [137]	PS, 203.89 ± 27.34	ICR mice	5 or 15 mg/kg/day, oral, 20 weeks	1. ↑ ER stress 2. ↑ ATF4-PPARγ/SREBP-1 pathway activation 3. ↑ Expression of lipid synthesis-related genes 4. ↑ Hepatic lipid metabolism disorder (↑ TC/LDL-C, ↓ serum HDL-C, ↑ serum TG, lipid droplet accumulation)
Chen et al., 2024 [139]	PS, 80	BALB/c mice	0.015 mg/day, 0.15 mg/day or 1.5 mg/day, oral, 21 days	1. Intestinal inflammation with impaired tight junction protein expression and ↑ serum LPS 2. ↑ Hepatic TLR4/NF-κB/NLRP3/GSDMD signaling 3. ↑ Hepatic inflammatory cytokines and hepatocyte pyroptosis
Lu et al., 2024 [140]	PS, 100 and 500	C57BL/6 mice	0.1 g/kg, oral, 180 days	1. ↑ TXNIP/NLRP3/GSDMD pathway activation 2. ↑ Hepatic pyroptosis (stronger effect of 100 nm than 500 nm PS-NPs) 3. Reversibility of NP-induced hepatic pyroptosis after 50-day recovery
Lin et al., 2024 [145]	PS, 200	Specific-pathogen-free (SPF) C57BL/6J mice	50 mg/kg/day, oral, 30 days	1. Gut microbiota dysbiosis, with stronger effects for NPs than for 100 μm MPs 2. ↑ Colonic TLR2-MyD88-NF-κB pathway activation 3. Liver damage with ballooning degeneration and oxidative stress
Fan et al., 2024 [149]	PS, 69 ± 2	ICR mice	15 mg/kg, oral, 2 weeks	1. ↑ Oxidative stress and inflammatory signaling 2. ↑ Insulin resistance signaling and ↓ hepatic insulin sensitivity 3. ↑ Lipid accumulation and lipogenesis through ERK-PPARγ/SREBP-1/ACC-1-related signaling
Lu et al., 2024 [155]	PS, 100 and 500	C57BL/6 mice	0.1 g/kg, oral (chow), 180 days	1. ↑ Hepatic lipid accumulation; stronger effect with 100 nm compared to 500 nm NPs 2. ↑ Autophagosome formation 3.↓ Lysosomal function and impaired autophagic flux
Zhu et al., 2023 [157]	PS, 100	*Monopterus albus*	0.05%, 0.5% and 1%, oral, 28 days	1. ↑ Disruption of hepatic glycolipid metabolism 2. ↑ PPAR-related metabolic signaling 3. ↑ Oxidative stress in the high-concentration group 4. ↑ Inflammatory response, MAPK signaling, and apoptosis in the hepatopancreas
Zhao et al., 2025 [171]	PS, 100	SPF grade C57BL/6 mice with colitis	10 mg/kg⋅b.w., oral, 5 weeks	1. ↑ Hepatic inflammation and metabolic dysregulation in mice with colitis 2. Dietary intervention with *Lactiplantibacillus plantarum* ZP-6 reduced hepatic NP retention and inflammation 3. ZP-6 intervention improved liver histopathology
Chi et al., 2022 [172]	PS, 100	C57BL/6 mice	5 μg/g⋅b.w., intraperitoneal injection, every 2 days for 2 weeks	1. ↑ ROS-NLRP3 axis activation 2. ↑ CCL2, ↑ CXCL12 3. ↑ Local neutrophil infiltration and ↑ NET formation 4. ↑ Hepatic inflammation
Wei et al., 2024 [173]	PS, 70	C57BL/6J mice (normal chow diet and HFD)	80 μg/g, oral, 26 weeks	1. ↑ Progression from HFD-induced steatosis to NASH 2. ↑ Hepatic steatosis and lipid uptake-related gene expression, especially under HFD 3. ↑ Hepatic steatosis (mice on HFD) 4. ↑ ER-mitochondria contacts 5. ↓ Nrf2/miR26a signaling (strongest functional effect in HFD mice) 6. ↑ Inflammation, and fibrosis (mice on HFD) 7. ↑ Vdac1 and ↑ Keap1 (most relevant in HFD mice)
Li et al., 2026 [78]	PS, 70	C57 BL/6J mice	50 mg/kg, oral gavage, 8 days	1. BA and ASBT-mediated NPs absorption 2. ↓ Lysosome biogenesis in the liver 3. ↓ CYP7A1 degradation 4. ↑ Hepatic bile acid synthesis 5. ↑ Colitis susceptibility of mice by reducing Lactobacillus and increasing Enterobacteriaceae 6. ↑ Male susceptibility to hepatic NPs effects (higher intestinal ASBT expression)
			50 mg/L, oral (water), 70 day	

* Arrows indicate the direction of change (↑ increase; ↓ decrease). ACC-1, acetyl-CoA carboxylase 1; ASBT, apical sodium-dependent bile acid transporter; ATF4, activating transcription factor 4; BA, bile acid(s); BMDMs, bone marrow-derived macrophages; b.w., body weight; CAT, catalase; CCL2, C-C motif chemokine ligand 2; CYP7A1, cholesterol 7 alpha-hydroxylase; CXCL12, C-X-C motif chemokine ligand 12; DRP1, dynamin-related protein 1; ER, endoplasmic reticulum; ERK, extracellular signal-regulated kinase; GSDMD, gasdermin D; GSH, glutathione;HDL-C, high-density lipoprotein cholesterol; HFD, high-fat diet; HO-1, heme oxygenase-1; IL-1β, interleukin 1 beta;Keap1, Kelch-like ECH-associated protein 1; LDL-C, low-density lipoprotein cholesterol; LPS, lipopolysaccharide; MAPK, mitogen-activated protein kinase; miR26a, microRNA-26a; mtROS, mitochondrial reactive oxygen species; MyD88, myeloid differentiation primary response 88; NASH, non-alcoholic steatohepatitis; NET, neutrophil extracellular trap; NF-κB, nuclear factor kappa B; NLRP3, NOD-like receptor family pyrin domain containing 3; NPs, nanoplastics/nanoparticles; NRF2/Nrf2, nuclear factor erythroid 2-related factor 2; OPA1, optic atrophy 1; PGC-1α, peroxisome proliferator-activated receptor gamma coactivator 1-alpha; p-NRF2, phosphorylated nuclear factor erythroid 2-related factor 2; PPAR, peroxisome proliferator-activated receptor; PPARγ, peroxisome proliferator-activated receptor gamma; PS, polystyrene; PS-NPs, polystyrene nanoplastics; ROS, reactive oxygen species; SREBP-1, sterol regulatory element-binding protein 1; TC, total cholesterol; TG, triglycerides; TLR2, toll-like receptor 2; TLR4, toll-like receptor 4; TXNIP, thioredoxin-interacting protein; Vdac1, voltage-dependent anion channel 1.

**Table 4:** Selected studies on the effects of MPs on the liver.

Reference	Type of Microplastic, Size [μm]	Experimental Model	Dose, Route of Exposure, Exposure Duration	Main Outcomes After Microplastic Exposure *
Zou et al., 2023 [124]	PS, 5 and 0.5	C57BL/6J mice	10 mg/L, oral (water), 3 months	1. ↓ Body weight 2. Disruption of liver architecture (↑ nuclear crinkling and ↑ mitochondrial damage) 3. ↑ Oxidative stress in hepatocytes 4. ↓ Antioxidant proteins (SIRT3, SOD2)
Li et al., 2025 [127]	PS, 0.5	ApoE^−/−^ mice	10 mg/L, oral (water), 12 weeks	1. ↑ Steatosis, fibrosis, and NAFLD severity (greater under WD). 2. ↑ Hepatic inflammation 3. ↓ Antioxidant capacity and ↑ lipid peroxidation 4. ↑ Lipogenesis and ↓ FA oxidation
Jeyavani et al., 2023 [128]	PP, 11.86–44.62	*Oreochromis mossambicus*	100 mg/kg, 500 mg/kg or 1000 mg/kg, oral, 96 h or 14 days	1. Dose- and time-dependent toxicity, greater effects after sub-acute exposure 2. ↑ ROS production and oxidative stress 3. Altered antioxidant enzyme activity 4. ↑ lipid peroxidation 5. ↑ apoptosis and DNA damage
Del Piano et al., 2024 [129]	PS, 1–20	Gilthead Seabreams (*Sparus aurata*)	25 mg/kg b.w./day or 250 mg/kg b.w./day, oral, 21 days	1. Dose-dependent hepatic inflammation, necrosis, and steatosis-like changes 2. ↑ Lipogenesis and lipid storage, with no major change in lipid catabolism genes 3. Impaired antioxidant defense and oxidative stress 4. Altered detoxification enzyme activity, mainly at the higher dose
Cheng et al., 2024 [6]	PP non-aged, 6.15 ± 2.01 PP aged, 5.01 ± 1.18 4.02 ± 1.26 3.96 ± 1.15	Balb/c mice	200 particles/mL oral, 28 days	1. Progressive aging-dependent effects of PP 2. ↑ Reductive stress (↑ NADH/NAD^+^ ratio) 3. Mitochondrial dysfunction 4. Disruption of nutrient transporters and NADH-related gene expression 5. Hepatotoxicity, systemic metabolic alterations and ↓ body weight gain
Xu et al., 2024 [142]	PE, 5	C57BL/6 mice	1 mg/L or 10 mg/L, oral (water), 21 days	1. Disruption of gut microbiota homeostasis (shift toward pathogenic microbiota and reduced beneficial bacteria) 2. ↑ Oxidative stress, inflammation, apoptosis, and necrosis in the liver 3. ↑ TLR2/NF-κB/NLRP3 pathway activation 4. Liver injury associated with gut–liver axis dysregulation
Djouina et al., 2023 [147]	PE, 10–45 and 106–125	C57BL/6 mice	100 µg/g, oral, 6 and 9 weeks	1. ↑ Fatty acid uptake and lipogenesis 2. Limited changes in β-oxidation and triglyceride synthesis 3. ↓ Detoxification pathways and ↑ oxidative imbalance 4. ↑ Hepatic inflammation, hepatocellular proliferation and fibrogenesis
Li et al., 2025 [150]	PS, 1	C57BL/6N mice short-term biodistribution study	0.2 mg, oral, 24 h	Short-term study: 1. MPs accumulation in the liver and testes Long-term exposure experiment: 1. ↑ Body fat percentage, without changes in food intake (after 9–12 weeks) 2. ↑ Hepatic lipid deposition 3. ↑ Lipogenesis with ↓ lipid oxidation and ↓ lipid transport 4. Kupffer cell polarization imbalance: ↑ M1, ↓ M2 phenotype 5. ↑ IL-17–NF-κB/MAPK signaling pathways 6. ↓ Anti-inflammatory cytokines
		C57BL/6N mice long-term exposure experiment	1 mg/L or 5 mg/L, oral, 3, 6, 9 or 12 weeks	
Zhang et al., 2025 [156]	PS, 5	Japanese quail (*Coturnix japonica*)	0.02 mg/kg, 0.4 mg/kg, 8 mg/kg, oral, 5 weeks	1. ↑ Hepatic lipid accumulation 2. Liver microstructural and ultrastructural damage 3. ↑ Oxidative stress 4. ↑ Inflammation/apoptosis pathways, including MAPK, TLR, FoxO)
Boran et al., 2024 [159]	PMMA, 3–10	HepG2/THP-1 co-culture	0.25–1 mg/mL, 72 h	1. ↓ Hepatocyte viability (stronger effect in HepG2 than THP-1) 2. ↑ Oxidative stress: (co-culture) 3. ↑ Pro-inflammatory cytokine secretion (co-culture) 4. ↓ β-oxidation; ↑ lipogenesis and lipid uptake; altered cholesterol metabolism; biphasic fatty acid transport response (HepG2 in co-culture) 5. ↑ Mitochondrial damage and lipid droplet accumulation (HepG2)
Najahi et al., 2025 [160]	PE 1–2.6 PET, <1 and 1–2.6	HepG2	10 µg/mL, 72 h	1. ↑ Cell viability 2. ↑ Oxidative stress 3. Mitochondrial dysfunction with the strongest effect observed for 2.6 μm PET particles 4. Activation of autophagy with the strongest observed for 1 μm PET particles
Ji et al., 2025 [166]	PET, 1	Balb/c mice	0.01 mg/day, 0.1 mg/day or 1 mg/day, oral, 42 days	1. ↓ Body weight gain 2. Liver injury with histopathological changes (hepatocyte swelling, inflammatory infiltration, collagen deposition) 3. Hepatotoxity (↑ ALT, ↑ AST) 4. ↑ Oxidative stress markers 5. ↑ p38 MAPK/p65 NF-κB signaling pathway
Li et al., 2021 [174]	PS, 5	C57 mice	20 mg/kg/day, oral, 30 days	1. ↑ Hepatic inflammation, apoptosis, and oxidative stress 2. ↑ Nrf2 expression without changes in HO-1 and NQO1 levels
Kaloyianni et al., 2021 [175]	PS, 5–12	Zebrafish (*Danio rerio*)	10 mg/g, oral, 21 days	1. ↑ Hepatic lipid peroxidation: ↑ MDA in *P. fluviatilis*; no significant change in *D. rerio* 2. ↑ Hepatic protein carbonylation in both species, more pronounced in *P. fluviatilis* 3. ↑ Hepatic genotoxicity in both species 4. ↑ Hepatic apoptosis and autophagy in both species 5. ↑ Hepatic amino acid and energy metabolism disruption: more pronounced in *P. fluviatilis* 6. ↑ Species-specific hepatic susceptibility: greater overall hepatic sensitivity in *P. fluviatilis* than in *D. rerio*
		*Perca fluviatilis*	134 mg/g, oral, 21 days	
Zhao et al., 2021 [176]	PS, 0.5	C57BL/6J mice	0.5 mg/day, oral, 4 weeks	1. Hepatotoxity (↑ ALT, ↑ AST) 2. Altered immune cell infiltration in liver (↑ NK cells and macrophages; ↓ B cells) 3. ↑ Pro-inflammatory cytokines. 4. ↓ Anti-inflammatory cytokines 5. ↑ NF-κB pathway activation in hepatic non-parenchymal cells
Li et al., 2024 [177]	unspecified, 0.5	db/m mice and db/db mice	1 mg/L, oral (water), 3 months	Changes in the db/db + MPs group relative to untreated db/db mice; no MP-exposed db/m group was included: 1. ↓ Body weight 2. ↑ Blood glucose and impaired glucose tolerance and ↑ hepatic glycogen accumulation 3. ↑ Hepatic gluconeogenesis (PP2A/AMPK/HNF4A pathway disruption) 4. ↑ Oxidative stress and redox imbalance 5. Dysregulated lipid metabolism (↑ lipogenesis, ↓ catabolism) 6. ↑ Liver fibrosis
Wen et al., 2024 [178]	PS, 5	C57BL/6 mice	0.05, 0.5, and 5 mg/kg body weight/day, oral, 30 days	1. Intestinal barrier damage and ↑ hepatic MP accumulation (mice) 2. Liver injury associated with dysregulation of bile acid metabolism (mice) 3. Altered expression of BA synthesis and efflux genes and cholestasis (mice; BA-related gene expression also confirmed in HepG2 cells) 4. Gut microbiota alterations and changed primary/secondary BA ratio. (mice) 5. ↑ Disruption of gut–liver BA circulation (gut–liver axis dysfunction) (mice)
		HepG2 cells	100, 200, 400 and 800 μg/mL	
Li et al., 2024 [179]	PS, 1	Human hepatobiliary organoids (HBOs)	2.5 μg/mL, 48 h	1. Hepatotoxicity and hepatocyte damage 2. Accumulation of MPs in bile ducts of hepatobiliary organoids 3. Disruption of bile acid metabolism 4. MP transport mediated by bile transporters (BSEP and MRP-2; dose-dependent)

* Arrows indicate the direction of change (↑ increase; ↓ decrease). ALT, alanine aminotransferase; AMPK, AMP-activated protein kinase; AST, aspartate aminotransferase; BA, bile acid(s); BSEP, bile salt export pump; b.w., body weight; FA, fatty acid; FoxO, forkhead box O; HNF4A, hepatocyte nuclear factor 4 alpha; HO-1, heme oxygenase 1; IL-17, interleukin 17; MAPK, mitogen-activated protein kinase; MDA, malondialdehyde; MP/MPs, microplastic(s); MRP-2, multidrug resistance-associated protein 2; NADH/NAD^+^, reduced nicotinamide adenine dinucleotide/oxidized nicotinamide adenine dinucleotide; NAFLD, non-alcoholic fatty liver disease; NF-κB, nuclear factor kappa B; NK cells, natural killer cells; NQO1, NAD(P)H quinone dehydrogenase 1; NLRP3, NOD-like receptor family pyrin domain containing 3; Nrf2/NRF2, nuclear factor erythroid 2-related factor 2; PE, polyethylene; PET, polyethylene terephthalate; PMMA, polymethyl methacrylate; PP, polypropylene; PP2A, protein phosphatase 2A; PS, polystyrene; ROS, reactive oxygen species; SIRT3, sirtuin 3; SOD2, superoxide dismutase 2; TLR, toll-like receptor; TLR2, toll-like receptor 2; WD, Western diet.

## Data Availability

No new data were created or analyzed in this study.

## Abbreviations

The following abbreviations are used in this manuscript:

8-OHdG	8-hydroxy-2′-deoxyguanosine
ACC1	acetyl-CoA carboxylase 1
ACOX1	acyl-CoA oxidase 1
ACSL1	long-chain acyl-CoA synthetase 1
AFM	atomic force microscopy
AFM-IR	atomic force microscopy–infrared spectroscopy
AKT	protein kinase B
aMPs	aged microplastics
AML-12	alpha mouse liver 12 cell line
AMPK	AMP-activated protein kinase
ANGPTL4	angiopoietin-like 4
ATF4	activating transcription factor 4
ATG5	autophagy related 5
ATG12	autophagy related 12
ATI	alveolar type I
ATII	alveolar type II
ATP	adenosine triphosphate
Bax	BCL2-associated X protein
Bcl-2	B-cell lymphoma 2
BPA	bisphenol A
Caco-2	human epithelial colorectal adenocarcinoma cell line
CAT	catalase
Ccl2	mouse gene encoding C-C motif chemokine ligand 2
CCL2	C-C motif chemokine ligand 2
Ccl5	C-C motif chemokine ligand 5
CDAHFD	choline-deficient, L-amino acid-defined, high-fat diet
CHOP	C/EBP homologous protein
CTB-gold	cholera toxin B-gold
Cu	copper
cGAS	cyclic GMP-AMP synthase
CPT1α	carnitine palmitoyltransferase 1 alpha
CYP	cytochrome P450
CYP7A1	cholesterol 7 alpha-hydroxylase
CXCL1	C-X-C motif chemokine ligand 1
CXCL2	C-X-C motif chemokine ligand 2
CXCL12	C-X-C motif chemokine ligand 12
DBP	dibutyl phthalate
DG	diacylglycerols
DLS	dynamic light scattering
DMSO	dimethyl sulfoxide
Drp1	dynamin-related protein 1
dsDNA	double-stranded DNA
ECM	extracellular matrix
EIF2α	eukaryotic initiation factor 2 alpha
EIPA	5-(N-ethyl-N-isopropyl)amiloride
EMT	epithelial–mesenchymal transition
ER	endoplasmic reticulum
FAE	follicle-associated epithelium
FABP1	fatty acid binding protein 1
FASN	fatty acid synthase
FGFR4	fibroblast growth factor receptor 4
FTIR	Fourier transform infrared spectroscopy
GSDMD	gasdermin D
GPx	glutathione peroxidase
GPX4	glutathione peroxidase 4
GST	glutathione S-transferase
HBOs	human hepatobiliary organoids
HepG2	human hepatocellular carcinoma cell line
Hg	mercury
Hh	Hedgehog
HL	hepatic lipase
HO-1	heme oxygenase-1
HT29-MTX	mucus-secreting HT29-MTX cell line
HuH-7	human hepatoma cell line
H_2_O_2_	hydrogen peroxide
IL-1β	interleukin 1 beta
IL-6	interleukin 6
IL-10	interleukin 10
IL-17A	interleukin 17A
IL-18	interleukin 18
JNK	c-Jun N-terminal kinase
KOH	potassium hydroxide
Keap1	Kelch-like ECH-associated protein 1
L02	human normal liver cell line
LC3II	microtubule-associated protein 1A/1B-light chain 3 II
LD-IR	laser infrared spectroscopy
LPS	lipopolysaccharide
MAFLD	metabolic dysfunction-associated fatty liver disease
MAMs	mitochondria-associated membranes
MCC	mucociliary clearance
MCP-1	monocyte chemoattractant protein 1
MDA	malondialdehyde
MeHg	methylmercury
METs	macrophage extracellular traps
miR-26a	microRNA-26a
MNPs	micro- and nanoplastics
MUC2	mucin 2
mtDNA	mitochondrial DNA
mTOR	mechanistic target of rapamycin
NAFL	non-alcoholic fatty liver
NAFLD	non-alcoholic fatty liver disease
NASH	non-alcoholic steatohepatitis
NETs	neutrophil extracellular traps
NF-κB	nuclear factor kappa B
NLRP3	NOD-like receptor family pyrin domain containing 3
NPs	nanoplastics
NQO1	NAD(P)H:quinone oxidoreductase 1
Nrf2	nuclear factor erythroid 2-related factor 2
NTA	nanoparticle tracking analysis
O-PTIR	optical photothermal infrared spectroscopy
OPA1	optic atrophy 1
PA	phosphatidic acids
P2X7	P2X purinoceptor 7
PBDEs	polybrominated diphenyl ethers
PCK	phosphoenolpyruvate carboxykinase
PDGF	platelet-derived growth factor
PDGFRα	platelet-derived growth factor receptor alpha
PE	polyethylene
PE-MPs	polyethylene microplastics
PERK	protein kinase R-like endoplasmic reticulum kinase
PET	polyethylene terephthalate
PET-MPs	polyethylene terephthalate microplastics
PET-NPs	polyethylene terephthalate nanoplastics
p-Drp1	phosphorylated dynamin-related protein 1
PCL	periciliary layer
PGC-1α	peroxisome proliferator-activated receptor gamma coactivator 1-alpha
PLA	polylactic acid
PLA-NPs	polylactic acid nanoplastics
PMMA	polymethyl methacrylate
POPs	persistent organic pollutants
PP	polypropylene
PPARα	peroxisome proliferator-activated receptor alpha
PPARγ	peroxisome proliferator-activated receptor gamma
PS	polystyrene
PS-MPs	polystyrene microplastics
PSNPs	polystyrene nanoparticles
PU	polyurethane
PUFAs	polyunsaturated fatty acids
PVC	polyvinyl chloride
PVC-MPs	polyvinyl chloride microplastics
Py-GC-MS	Pyrolysis–gas chromatography–mass spectrometry
ROS	reactive oxygen species
RXR	retinoid X receptor
SEM	scanning electron microscopy
SIM	selected ion monitoring
sIgA	secretory immunoglobulin A
SIRT3	sirtuin 3
SLC7A11	solute carrier family 7 member 11
SOD	superoxide dismutase
SOD2	superoxide dismutase 2
SREBP1	sterol regulatory element-binding protein 1
STING	stimulator of interferon genes
TEM	transmission electron microscopy
TGF-β	transforming growth factor beta
THP-1	human monocytic cell line
TiO2	titanium dioxide
TLR	Toll-like receptor
TLR2	Toll-like receptor 2
TLR4	Toll-like receptor 4
TNF-α	tumor necrosis factor alpha
TRIB3	tribbles pseudokinase 3
UPR	unfolded protein response
UV	ultraviolet
ZnCl_2_	zinc chloride
ΔΨm	mitochondrial membrane potential
